# The miRNA‐mRNA Regulatory Network in Human Hepatocellular Carcinoma by Transcriptomic Analysis From GEO

**DOI:** 10.1002/cnr2.70098

**Published:** 2025-01-07

**Authors:** Razieh Heidari, Vahideh Assadollahi, Seyedeh Negar Marashi, Fatemeh Elahian, Seyed Abbas Mirzaei

**Affiliations:** ^1^ Cancer Research Center, Basic Health Sciences Institute Shahrekord University of Medical Sciences Shahrekord Iran; ^2^ Department of Medical Biotechnology, School of Advanced Technologies Shahrekord University of Medical Sciences Shahrekord Iran; ^3^ Department of Tissue Engineering & Applied Cell Sciences, School of Advanced Technologies Shahrekord University of Medical Sciences Shahrekord Iran; ^4^ Advanced Technology Cores Baylor College of Medicine Texas USA; ^5^ Cellular and Molecular Research Center, Basic Health Sciences Institute Shahrekord University of Medical Sciences Shahrekord Iran

**Keywords:** bioinformatics, biomarker, HCC, hepatocellular carcinoma, microarray, microRNA

## Abstract

**Background:**

Bioinformatics analysis of hepatocellular carcinoma (HCC) expression profiles can aid in understanding its molecular mechanisms and identifying new targets for diagnosis and treatment.

**Aim:**

In this study, we analyzed expression profile datasets and miRNA expression profiles related to HCC from the GEO using R software to detect differentially expressed genes (DEGs) and differentially expressed miRNAs (DEmiRs).

**Methods and results:**

Common DEGs were identified, and a PPI network was constructed using the STRING database and Cytoscape software to identify hub genes. The reduced levels of tumor suppressor miRNAs or down regulated DEmiRs may be increased levels of oncogenes, the oncomirs or up regulated DEmiRs may be decreased levels of tumor suppressor genes in cancerous cells. According to this strategy, increased and decreased DEGs, also increased and decreased DEmiRs were selected. The multimir package was employed to predict target genes for DEmiRs then DEmiRs‐hub gene network created. We identified approximately 1000 overlapping DEGs and 60 DEmiRs. Hub genes included RRM2, *MELK*, *KIF11*, *KIF23*, *NCAPG*, *DLGAP5*, *BUB1B*, *AURKB*, *CCNB1*, *KIF20A*, *CCNA2*, *TTK*, *PBK*, *TOP2A*, *CDK1*, *MAD2L1*, *BIRC5*, *ASPM*, *CDCA8*, and *CENPF*, all associated with significantly worse survival in HCC. miR‐224, miR‐24, miR‐182, miRNA‐1‐3p, miR‐30a, miR‐27a, and miR‐214 were identified as important DEmiRs with targeting more than six hub genes.

**Conclusion:**

Generally, our findings offer insight into the interaction of hub genes and miRNAs in the development of HCC by bioinformatics analysis, information that may prove useful in identifying biomarkers and therapeutic targets in HCC.

**Abbreviations:**
BP: biological process
CC: cellular component
CRC: colorectal cancer
DEGs: differentially expressed genes
DEmiR: differentially expressed miRNA
FC: fold‐change
GEO: gene expression omnibus
GO: gene ontology
HCC: hepatocellular carcinoma
HR: hazard ratio
KEGG: Kyoto encyclopedia of genes and genomes
lncRNA: long non‐coding RNA
MF: molecular function
miRNA: microRNA
ncRNA: non‐coding RNA
PPI: protein–protein interaction
STRING: search tool for interacting genes

## 1 Introduction

Cancer, a deadly disease, arises from disruptions in gene expression [1]. Hepatocellular carcinoma (HCC) is usually discovered when the cancer has progressed to an advanced stage, at which point there are few therapeutic choices [2]. Biopsy is necessary for detecting most malignancies, including HCC, although multidisciplinary approaches involving clinical, radiological, and laboratory modalities can aid in diagnosis. Treatment options for HCC include systemic chemotherapy, radiofrequency ablation, and molecular therapies, yet unsatisfactory clinical outcomes persist due to ineffective treatment, late diagnosis, and high rates of metastasis and recurrence [3, 4]. Therefore, identifying non‐invasive biomarkers and new therapeutic targets is crucial for early HCC detection and effective therapy [5, 6, 7]. Generally, differentially expressed genes (DEGs) between tumor and healthy tissue are regarded as the cancer stimulating genes [8]. Numerous studies have implicated dysregulation of mRNAs and miRNAs in liver carcinogenesis [7, 9, 10]. miRNAs regulate gene expression at transcriptional levels [11, 12]. miRNAs whose expression is elevated in malignancies may be defined as oncogenes. Known as oncomirs, these oncogenic miRNAs typically stimulate tumor growth by suppressing the expression of tumor suppressor genes. Conversely, in malignant cells, the expression of certain miRNAs is reduced. These miRNAs are regarded as tumor suppressor miRNAs. Typically, tumor suppressor miRNAs halt tumor growth by inhibiting oncogenes [13, 14]. Identifying differentially expressed miRNAs (DEmiR) between tumor and normal tissues can help pinpoint biomarkers in human malignancies. Previous studies have demonstrated the presence and stability of miRNAs in blood [15, 16].

Bioinformatics analysis has been playing a vital role to discover potential genomic biomarkers compared to the wet‐lab‐based experimental approaches, more correctly from a vast number of candidates by saving time and cost for disease diagnosis, prognosis, and therapies [17, 18]. For example *CDK1*, *AURKA*, *CDC20*, *CCNB2*, *TOP2A*, *PLK1*, *BUB1B*, and *BIRC5* were identified as the key genes in HCC by integrated bioinformatics that may provide valuable resources for HCC therapy [19]. Analysis of gene and miRNA expression profiles using NGS and microarray techniques has increased our understanding of HCC's molecular mechanisms [6, 20, 21]. Employing bioinformatics analysis and high‐throughput technologies can aid in identifying differentially expressed genes, proteins, biological pathways, and noncoding RNAs in tumors, potentially leading to the discovery of treatment targets or biomarkers in various human malignancies [7, 8, 22, 23, 24, 25].

**FIGURE 1:**
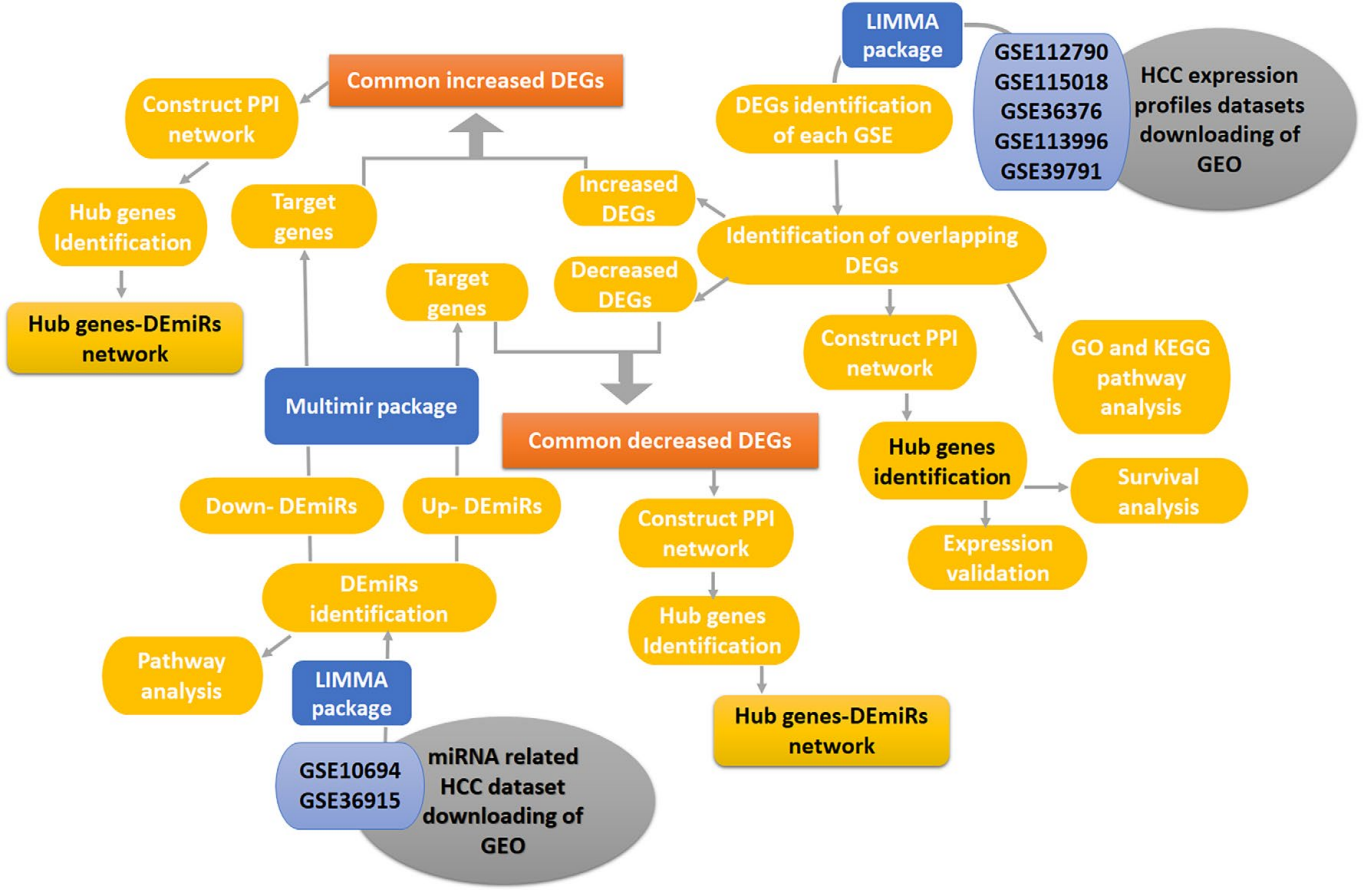
Flow diagram of data analysis GSE112790, GSE115018, GSE36376, GSE113996, GSE39791, GSE10694, and GSE36915 related HCC of the GEO (www.ncbi.nlm.nih.gov/geo).

In the present work, to detect DEGs and DEmiRs or DEMs in HCC, five gene microarray datasets (GSE112790, GSE115018, GSE36376, GSE113996, and GSE39791), along with miRNA expression datasets GSE10694 and GSE36915, were chosen from the GEO (Gene Expression Omnibus) database and analyzed using R software. After identifying DEGs and DEmiRs, hub genes were screened from the PPI network and the multimir package in R were used to predict target genes for DEmiR. The reduced levels of tumor suppressor miRNAs or down regulated DEmiRs may be increased levels of oncogenes, the oncomirs or up regulated DEmiRs may be decreased levels of tumor suppressor genes in cancerous cells. According to this strategy, DEmiRs‐hub gene network created to illustrate the dysregulated key DEmiRs with more interactions with the hub genes (Figure 1).

## 2 Methodology

### 2.1 Data Collection From GEO Repository

Five datasets (GSE112790, GSE115018, GSE36376, GSE113996, and GSE39791) were selected to identify key regulatory genes in HCC from the GEO database (www.ncbi.nlm.nih.gov/geo). Additionally, two miRNA‐related HCC datasets, GSE10694 and GSE36915, were downloaded from GEO. All datasets contained samples from HCC and adjacent tumor‐free tissues. The characteristics of the datasets, including the platform used and the number of healthy and cancerous samples, are provided in the Table 1.

**TABLE 1:** Details for selected datasets related to HCC of GEO.

GSE	Platform	Samples	Number of normal (noncancerous) sample	Number of tumor sample
GSE112790 (Public on Jan 03, 2019)	GPL570	198	15	183
GSE115018 (Public on May 30, 2018)	GPL20115	24	12	12
GSE36376 (Public on Mar 20, 2012)	GPL10558	433	193	240
GSE113996 (Public on May 03, 2021)	GPL16043	40	20	20
GSE39791 (Public on Nov 03, 2014)	GPL10558	144	72	72
GSE10694 (Public on Dec 31, 2008)	GPL6542	166	88	78
GSE36915 (Public on Apr 01, 2013)	GPL8179	89	21	68

### 2.2 Identification of DEGs and DEmiRs in HCC

The raw expression data from GSE112790, GSE115018, GSE36376, GSE113996, and GSE39791 were individually preprocessed using R v3.4.1 (https://www.r‐project.org/) to identify DEGs in HCC samples compared to tumor‐free tissue samples. For the detection of DEmiRs, GSE10694 and GSE36915 were analyzed. The quantile normalization technique was applied to obtain the normalized expression matrix of the datasets [26]. Boxplot diagrams were used to demonstrate the normalized data following the normalization procedure. The LIMMA package in bioconductor was utilized to identify DEGs or DEmiRs by comparing expression values between groups. Additionally, volcano plots were created using the Enhanced Volcano package in R. The cut‐off criteria of *p*‐value < 0.05 and |log2FC| > ± 1 for DEGs or DEmiRs were employed to determine significant DEGs or DEmiRs. To avoid omitting important genes, DEGs common to at least two or more databases were selected for further analysis. A Venn diagram was utilized to identify overlapping DEGs among the selected datasets. The Venn diagram was created using the online Venn diagram builder available at https://bioinformatics.psb.ugent.be/webtools/Venn.

The DEmiR were identified based on defined criteria *p*‐value < 0.05 and |log2FC| > ± 1 of the GSE10694 and GSE36915 analysis. In this study, the UALCAN database (http://ualcan.path.uab.edu) was utilized to validate the expression of top DEmiRs. UALCAN is an extensive and interactive online tool that analyzes canceromics data to identify biomarkers or validate potential genes of interest in silico [27].

### 2.3 PPI Networks of Overlapping DEGs in HCC

The STRING database (http://string.embl.de/) is a useful tool for constructing protein–protein interaction (PPI) networks to study how proteins interact functionally with each other [25, 26]. From the STRING biological database, the PPI network of the overlapping DEGs was extracted, and Cytoscape (version 3.7.2) was utilized to demonstrate it [27]. Moreover, the hub genes in the created network of DEGs were examined using the CytoHubba 0.1 plugin of Cytoscape, according to their MMC method‐calculated score.

### 2.4 Validating and Survival Analysis of Hub Genes Expression

A database specifically for cancer research, GEPIA database (http://gepia2.cancer‐pku.cn/) is intended to examine information from the genotype‐tissue expression (GTEx) and TCGA databases [28]. Here, the “Expression DIY” page of GEPIA was utilized to investigate and evaluate the expression of the hub genes between HCC and normal tissues. After selecting the liver hepatocellular cancer dataset and typing gene symbols into the text region, a comparative study between the tumor and normal samples was conducted using statistical analysis, with a threshold of *p*‐value < 0.01. The Kaplan–Meier plotter (http://kmplot.com/analysis) was then used to investigate the relationship between the genes' impact and patients' overall survival. This online tool can evaluate the prognostic values of genes in samples from various types of tumors [29]. The database can compute the log‐rank *p*‐value and the hazard ratio (HR) with a 95% confidence interval (95% CI). In this step, to identify statistically significant results, a *p*‐value of less than 0.05 was considered the significant threshold.

### 2.5 Enrichment Analysis of Overlapping DEGs

Utilizing gene ontology (GO) analysis in high‐throughput genome or transcriptome data enables evaluation for functional enrichment. The three primary functional categories of GO are biological process (BP), molecular function (MF), and cellular component (CC) [28]. Kyoto encyclopedia of genes and genomes (KEGG) pathway analysis and GO annotation were performed using FunRich software to further investigate the biological roles of common DEGs in HCC. The *p*‐value is used to filter the best results; a *p*‐value of less than 0.05 is considered statistically significant.

### 2.6 Pathway Analysis of the DEmiRs

The functional role of the DEmiRs was investigated through pathway analysis to identify important biochemical pathways controlled by selected miRNAs in tumor development using the bioinformatics tool DIANA‐mirPath v.3 (http://www.microrna.gr/miRPathv3). A *p*‐value < 0.05 was considered statistically significant when using DIANA‐mirPath.

### 2.7 Identification of the Target Gene for DEmiRs

To find the target genes of the identified DEmiRs, the Multimir package in R software was employed. This package includes eight predicted miRNA‐target databases, three disease‐ and drug‐related miRNA databases, and three validated miRNA‐target databases (miRecords, miRTarBase, and TarBase). The Multimir package constitutes an extensive collection of predicted and confirmed miRNA‐target relationships, along with their correlations to illnesses and medications [30, 31]. Target genes for upregulated DEmiRs or downregulated DEmiRs were predicted using the TarBase, miRecords, and miRTarBase databases through the Multimir package.

### 2.8 DEmiRs‐DEGs Network Construction in HCC

In cancer, reduced levels of tumor suppressor miRNAs or downregulated DEmiRs may lead to increased levels of oncogenes, while the increased expression of oncomirs may result in decreased expression levels of tumor suppressor genes in cancerous cells. Considering the importance of hub genes in PPI network regulation, DEmiRs that regulate these genes can be identified as key miRNAs in HCC. To identify the interaction between DEmiRs and DEGs in HCC, the first step involved selecting upregulated and downregulated DEGs among at least two or more datasets for further analysis. The overlapping genes between the predicted target genes of upregulated DEmiRs and downregulated DEGs were found using a Venn diagram. Subsequently, PPI networks of the obtained overlapping downregulated DEGs were generated using STRING and Cytoscape software. In addition, overlapping genes between the predicted targets of downregulated DEmiRs and upregulated DEGs were identified using a Venn diagram, and PPI networks of the obtained overlapping upregulated DEGs were produced using STRING and Cytoscape software. Hub genes in the PPI networks of upregulated DEGs and downregulated DEGs were determined by CytoHubba through obtaining gene node scores using the MCC and degree method [29]. DEmiRs that had more interactions with the hub genes were introduced as key miRNAs in HCC, and Cytoscape software was used to illustrate hub genes‐DEmiR networks.

## 3 Results

### 3.1 Identification of DEGs and DEmiRs in HCC Samples

**FIGURE 2:**
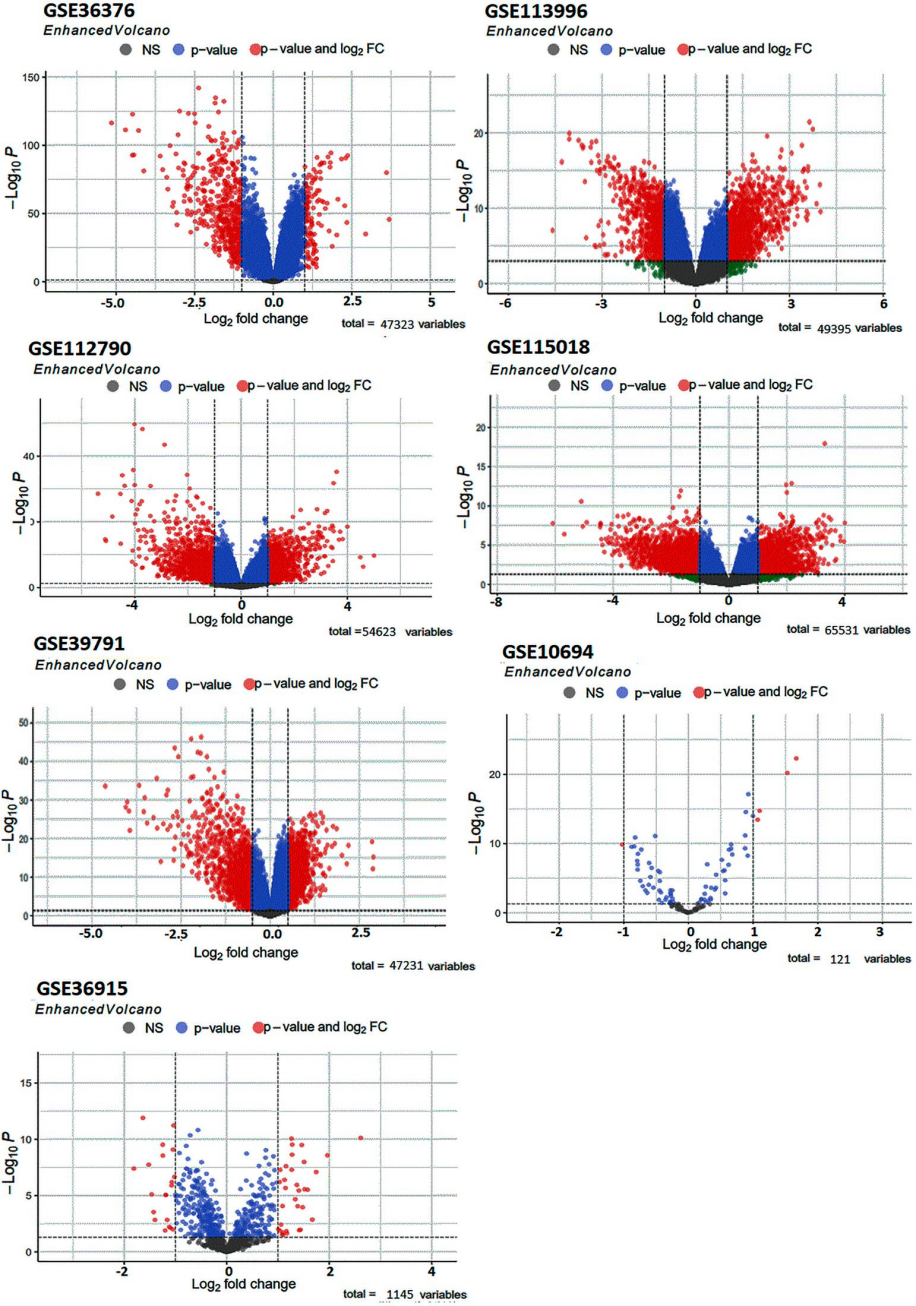
Volcano graphs for GSE112790, GSE115018, GSE36376, GSE113996, GSE39791, GSE10694, and GSE36915 dataset indicated the significant differences in expression between HCC and control samples based on *p*‐value < 0.05 and |log2FC| > 1. *p*‐value (blue dots), only logFC (green dots), both *p*‐value and log2FC (red dots), or not significant in both categories (gray dots) were indicated by colored dots. FC, fold‐change; HCC, hepatocellular carcinoma.

**FIGURE 3:**
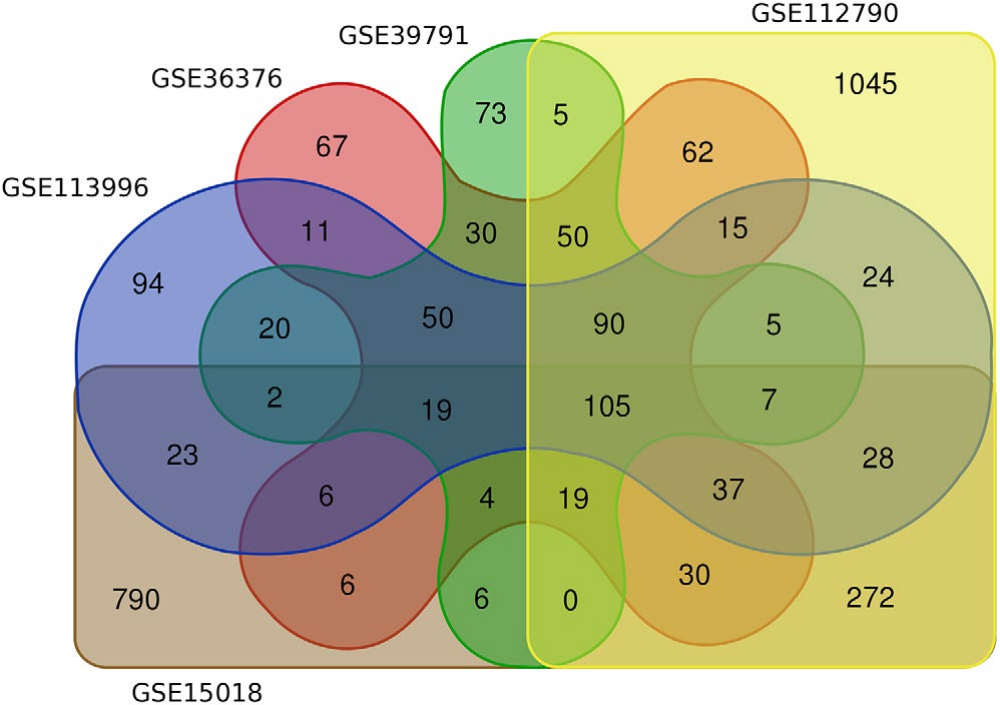
A Venn diagram (http://bioinformatics.psb.ugent.be/webtools/Venn) was created to illustrate the overlapping DEGs in GSE112790, GSE115018, GSE36376, GSE113996, and GSE39791 related to HCC. To ensure crucial genes were not overlooked and to comprehensively examine the obtained DEGs in HCC, common DEGs were selected from at least two or more datasets. DEGs, differentially expressed genes; HCC, hepatocellular carcinoma.

**FIGURE 4:**
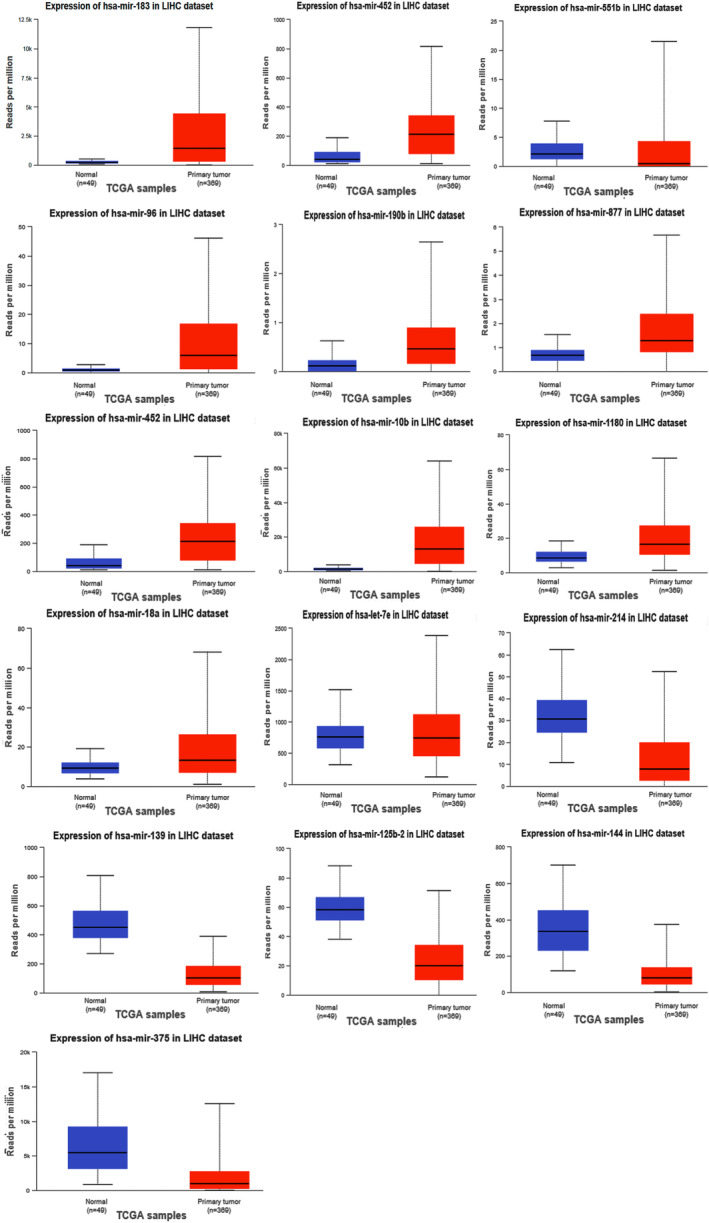
Expression levels of the top DEmiRs in TCGA normal (*n* = 49) and LIHC tumor (*n* = 369) were analyzed using the UALCAN database in HCC. The difference in miRNA expression between the cancer sample and the normal sample was significant in all investigated top DEmiRs, consistent with our analysis. However, the expression chart in the UALCAN database was not available for hsa‐miR‐886‐5p in LIHC. DEmiRs, differentially expressed miRNA; LIHC, liver hepatocellular carcinoma.

DEGs from tumor tissues compared with normal sample groups were obtained in HCC by analyzing expression profiles GSE112790, GSE115018, GSE36376, GSE113996, and GSE39791 from the GEO database. A total of 477 HCC and 347 healthy tissues were examined to identify DEGs. The volcano plots from each dataset were illustrated in Figure 2. To avoid missing critical genes and comprehensively examine the obtained DEGs in HCC, DEGs that were common in at least two or more databases were selected for further analysis. The Venn diagram identified approximately 1000 overlapping DEGs (Figure 3), including about 329 upregulated DEGs and about 698 downregulated DEGs. Analysis of GSE10694 and GSE36915 revealed about 60 DEmiRs in HCC tissue compared with normal samples, including 29 upregulated miRNAs and 24 downregulated miRNAs. Detailed information on the top DEmiRs, significantly upregulated, and downregulated miRNAs in HCC, was listed in Table 2. The expression of top DEmiRs, such as hsa‐miR‐183, hsa‐miR‐452, hsa‐miR‐886‐5p, hsa‐miR‐551b, hsa‐miR‐96, hsa‐miR‐10b, hsa‐miR‐214*, hsa‐miR‐139‐3p, hsa‐miR‐144*, and hsa‐miR‐125b‐2*, at UALKAN confirmed our results (Figure 4).

**TABLE 2:** Top DEmiRs (miRNA with increased and decreased expression) in HCC tissues compared with normal tissues were identified in analyses of GSE10694 and GSE36915 based on a *p*‐value < 0.05 and |log2FC| > 1.

Top DEmiRs	miRNA_ID	logFC	*p*	Adjusted *p*	Literature review
Increased DEmiRs	hsa‐miR‐183	2.706142	8.65E‐11	1.98E‐08	Upregulated in HCC [29, 32, 33]
	hsa‐miR‐452	2.026533	3.55E‐09	2.26E‐07	Upregulated in HCC [34, 35, 36, 37]
	hsa‐miR‐886‐5p	1.749826	1.01E‐07	3.13E‐06	Upregulated in HCC [36]
	hsa‐miR‐551b	1.667185	1.40E‐03	5.72E‐03	Upregulated in HCC [38, 39]
	hsa‐miR‐96	1.661249	2.76E‐06	3.72E‐05	Upregulated in HCC [40, 41, 42]
	hsa‐miR‐10b	1.576938	1.01E‐04	0.000691	Upregulated in HCC [43, 44, 45]
Decreased DEmiRs	hsa‐miR‐214*	−1.86906	3.75E‐08	1.53E‐06	A tumor suppressor in HCC [39, 46, 47]
	hsa‐miR‐139‐3p	−1.66415	5.58E‐13	6.39E‐10	A tumor suppressor in HCC [48, 49, 50]
	hsa‐miR‐144*	−1.54111	6.19E‐06	7.16E‐05	A tumor suppressor in HCC [50, 51]
	hsa‐miR‐125b‐2*	−1.53818	9.69E‐09	5.28E‐07	Down‐regulation in HCC [52, 53]

Abbreviations: DEmiR, differentially expressed miRNA; FC, fold‐change; HCC, hepatocellular carcinoma.

### 3.2 Construction of the PPI Network of Overlapping DEGs

**FIGURE 5:**
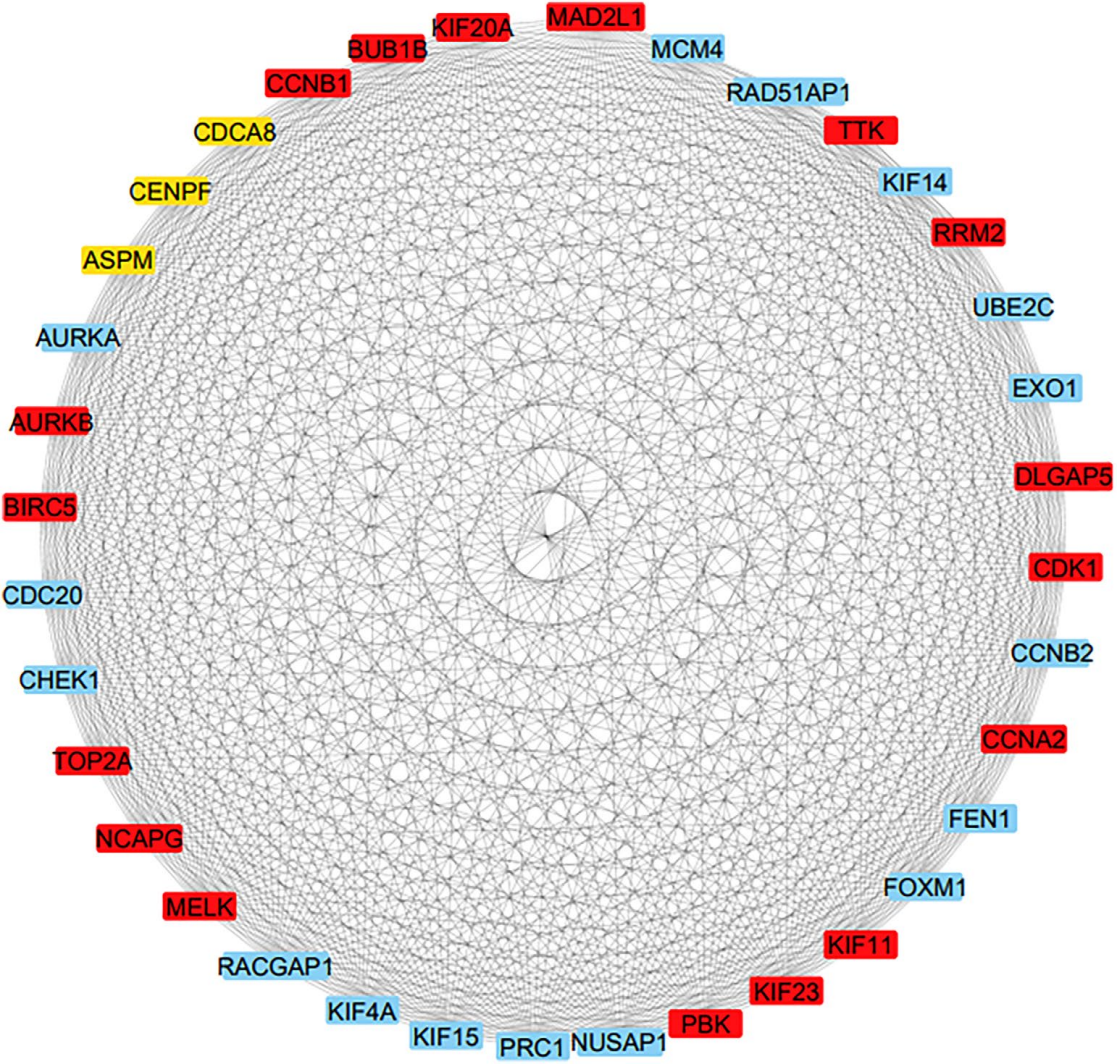
The PPI network of overlapping DEGs from the investigated datasets in HCC, comprising 837 nodes and 12 286 edges, was constructed through the STRING database and Cytoscape software. Within the hub genes, the red nodes represent those with higher node scores, while the yellow nodes represent those with lower node scores in the network.

To identify interactions between overlapping DEGs, we utilized approximately 1000 overlapping DEGs to construct a PPI network. The resulting network comprised 837 nodes and 12 286 edges. Subsequently, using the CytoHubba plugin, 20 hub nodes, including RRM2, MELK, KIF11, KIF23, NCAPG, DLGAP5, BUB1B, AURKB, CCNB1, KIF20A, CCNA2, TTK, PBK, TOP2A, CDK1, MAD2L1, BIRC5, ASPM, CDCA8, and CENPF, were identified using the MCC method. The significance of hub genes was depicted using a color spectrum ranging from red to yellow (Figure 5). The list of hub genes ranked by the MCC method is reported in Table 3.

**TABLE 3:** Hub genes in PPI Network of overlapping DEGs of the investigated datasets in HCC ranked using the CytoHubba plugin (MCC method) of the Cytoscape software.

Rank	Name	Score	Rank	Name	Score
1	RRM2	3.54E+75	1	CCNA2	3.54E+75
1	MELK	3.54E+75	1	TTK	3.54E+75
1	KIF11	3.54E+75	1	PBK	3.54E+75
1	KIF23	3.54E+75	1	TOP2A	3.54E+75
1	NCAPG	3.54E+75	1	CDK1	3.54E+75
1	DLGAP5	3.54E+75	16	MAD2L1	3.54E+75
1	BUB1B	3.54E+75	16	BIRC5	3.54E+75
1	AURKB	3.54E+75	18	ASPM	3.54E+75
1	CCNB1	3.54E+75	18	CDCA8	3.54E+75
1	KIF20A	3.54E+75	18	CENPF	3.54E+75

### 3.3 Validating and Survival Analysis of Hub Genes Expression in HCC

**FIGURE 6:**
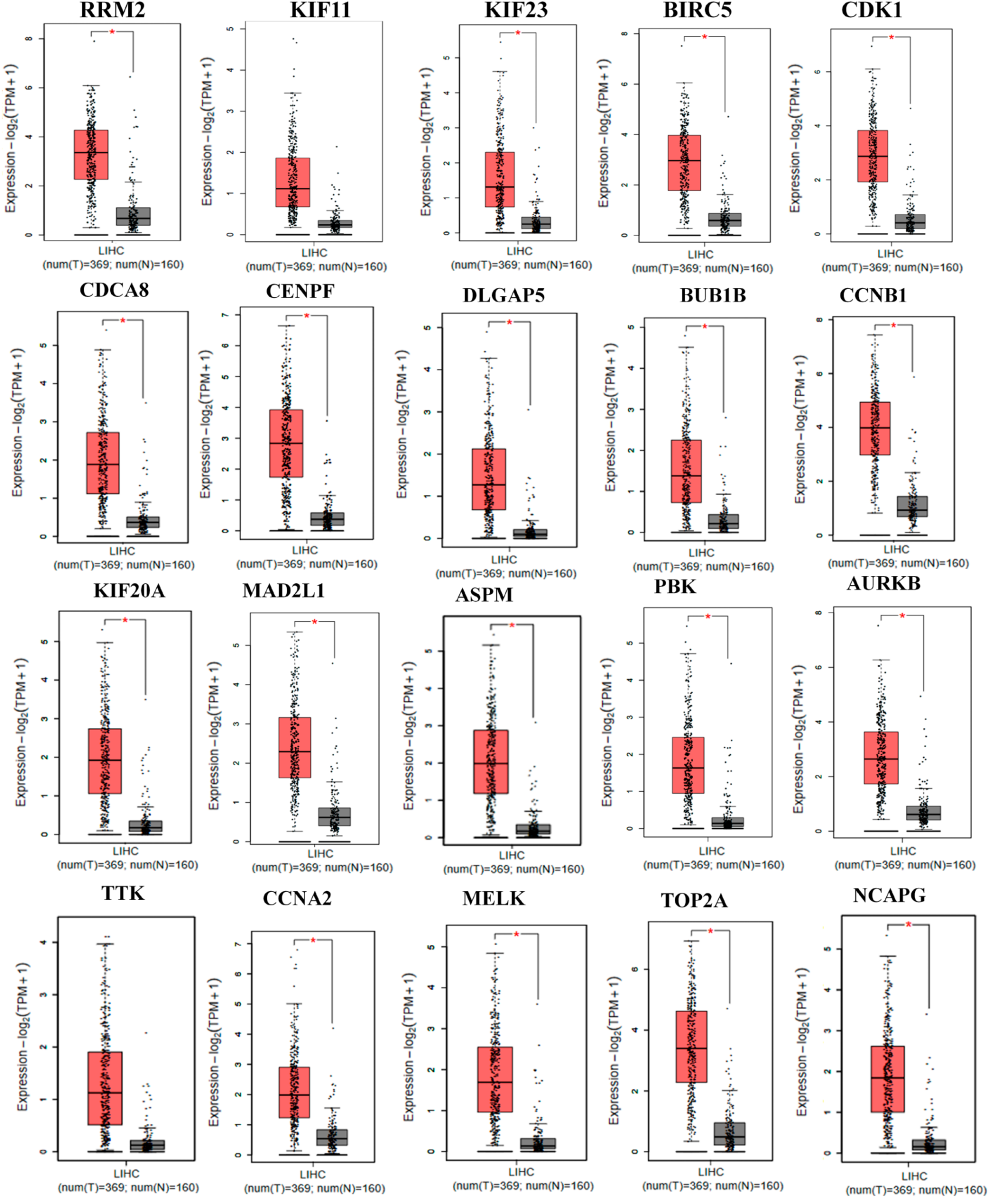
mRNA expression validation of identified hub genes from the overlapping DEGs analysis in HCC was conducted using the GEPIA database. Comparison of the expression levels of the 20 identified hub genes in HCC (red box; *n* = 369) and normal tissues (black box; *n* = 160) revealed that all hub genes except for KIF11 and DLGAP5 were significantly increased in HCC compared with normal liver tissues. A *p*‐value < 0.01 was considered statistically significant.

The mRNA expressions of all hub genes, namely RRM2, MELK, KIF23, NCAPG, BUB1B, AURKB, CCNB1, KIF20A, CCNA2, TTK, PBK, TOP2A, CDK1, MAD2L1, BIRC5, ASPM, CDCA8, and CENPF, except for KIF11 and DLGAP5, showed a significant increase in HCC compared with normal samples, as revealed by the GEPIA database. Although the expression of KIF11 and DLGAP5 exhibited an increasing pattern in GEPIA, this increase was not statistically significant (Figure 6). However, the expression patterns of KIF11 and DLGAP5 were significantly higher in HCC than in normal samples according to the UALCAN database.

**FIGURE 7:**
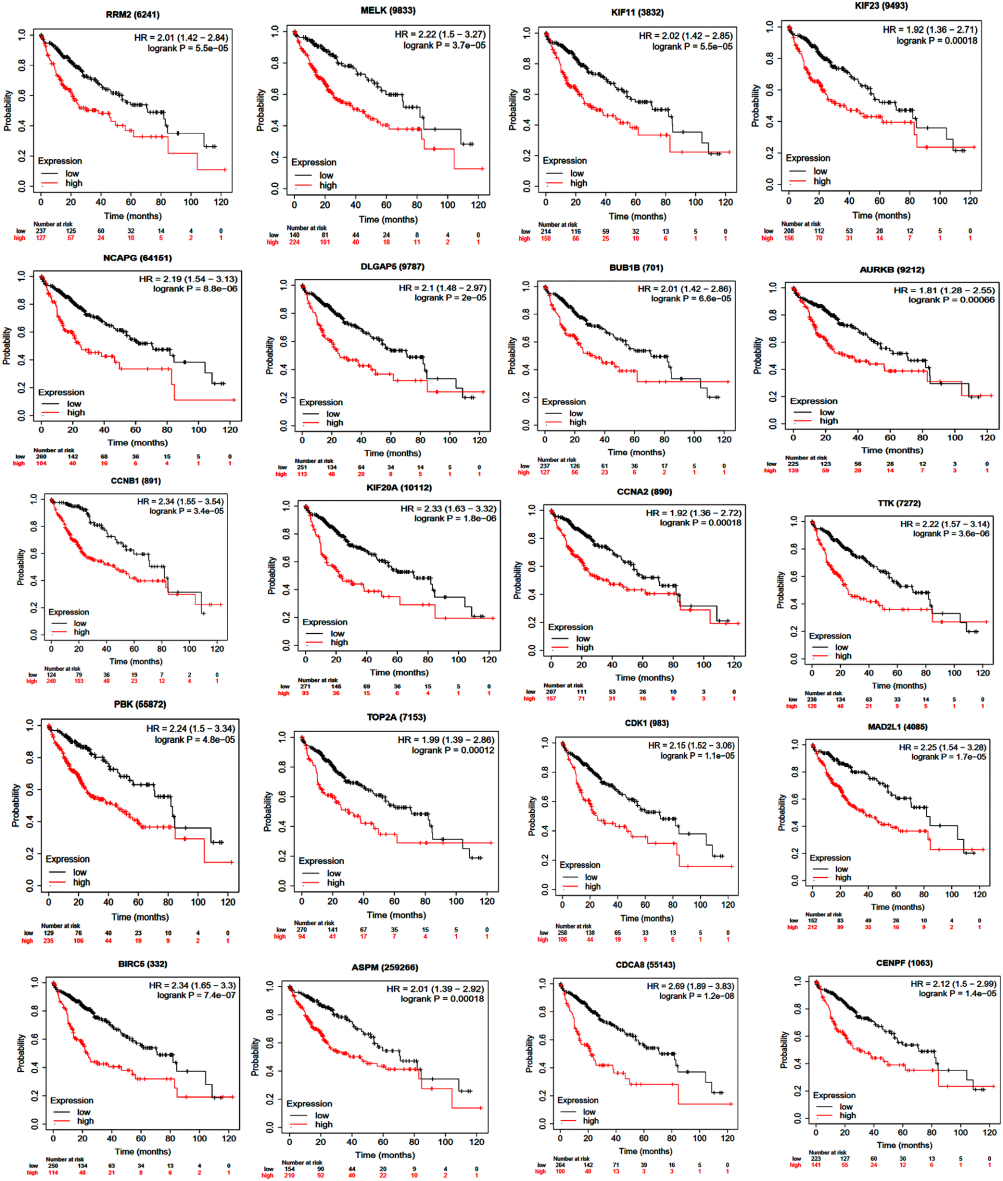
Kaplan–Meier overall survival analysis was conducted for the top 20 hub genes from the investigated datasets in HCC. High expression of RRM2, MELK, KIF23, NCAPG, BUB1B, AURKB, CCNB1, KIF20A, CCNA2, TTK, PBK, TOP2A, CDK1, MAD2L1, BIRC5, ASPM, CDCA8, CENPF, KIF11, and DLGAP5 was associated with poor overall survival of HCC patients. High expression of these hub genes was linked to unfavorable overall survival.

The Kaplan–Meier plotter can evaluate the impact of mRNA expression on survival in different tumors. To further validate candidate hub genes, their prognostic roles in HCC were examined using the Kaplan–Meier plotter database. As shown in Figure 7, high expression of RRM2, MELK, KIF23, NCAPG, BUB1B, AURKB, CCNB1, KIF20A, CCNA2, TTK, PBK, TOP2A, CDK1, MAD2L1, BIRC5, ASPM, CDCA8, CENPF, KIF11, and DLGAP5 indicated significantly unfavorable overall survival in patients with HCC.

### 3.4 Gene Enrichment and Pathway Analysis

**FIGURE 8:**
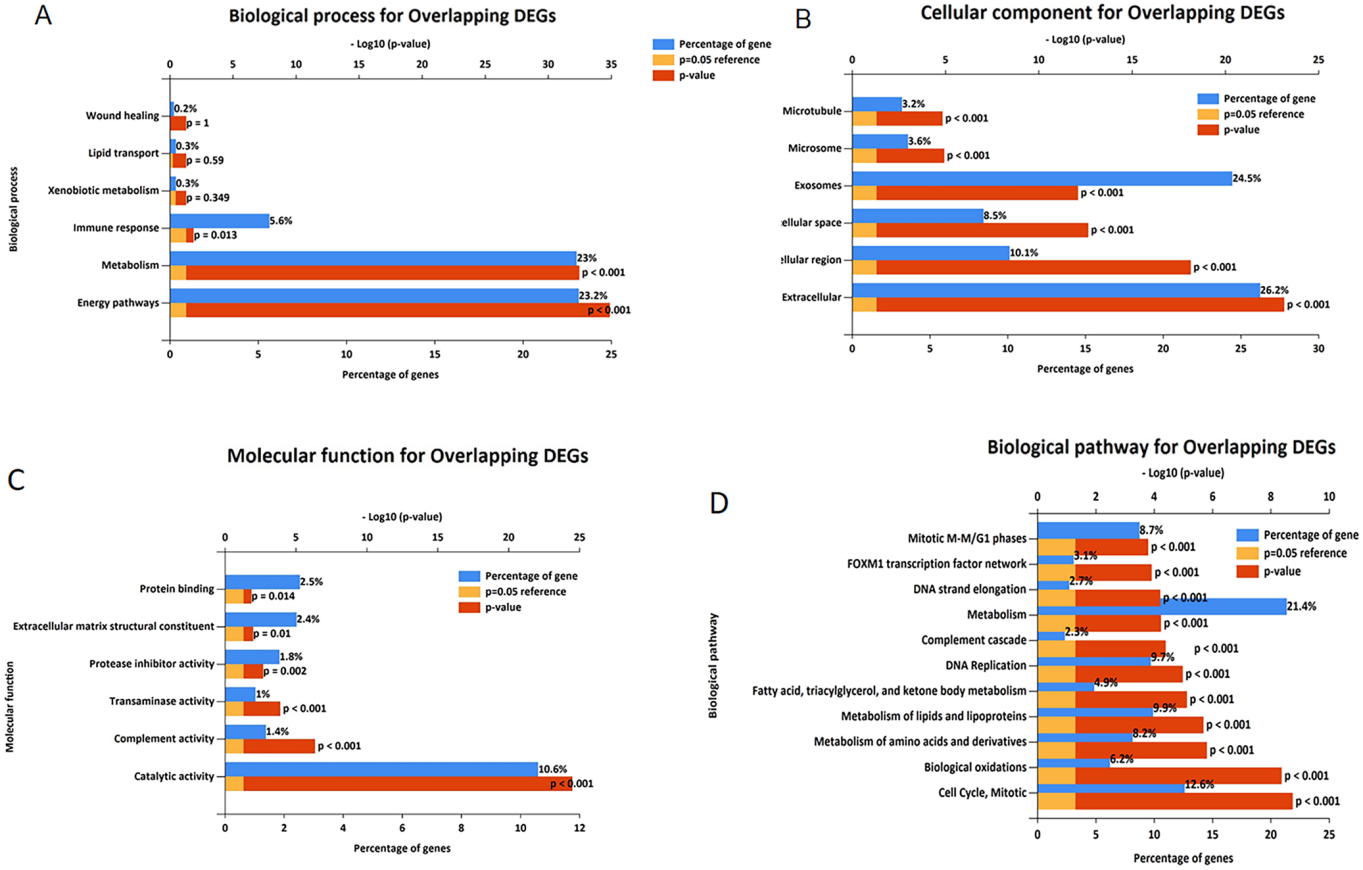
GO enrichment analysis was performed on the obtained overlapping DEGs from the investigated datasets in HCC to determine the most significant GO terms in (A) biological processes (BP), (B) cellular components (CC), and (C) molecular functions (MF) using FunRich software. (D) KEGG pathways for overlapping DEGs were discovered and visualized using FunRich software. Terms with a *p*‐value < 0.05 were considered significant.

GO and KEGG pathway analyses of the overlapping DEGs were conducted using FunRich software. The enrichment analysis of approximately DEGs revealed the most significant GO terms: in BP, energy pathways, metabolism, and immune response; in CC, extracellular and exosomes; and in MF, catalytic activity and complement activity. KEGG enrichment analysis for common DEGs mainly showed enrichment in cell cycle, mitotic processes, biological oxidations, DNA replication, complement cascade, metabolism, DNA strand elongation, and FOXM1 transcription. Other KEGG, BP, CC, and MF terms for DEGs are presented in Figure 8. The complete set of findings from the KEGG and GO pathway enrichment analysis is provided in the Supporting Information.

Based on the miRNA pathway analysis results, DEmiRs were particularly enriched in the TGF‐beta signaling pathway, MAPK signaling pathway, neurotrophin signaling pathway, glycosphingolipid biosynthesis—lacto and neolacto series, and Ras signaling pathway (Table 4).

**TABLE 4:** Main pathways for DEmiRs in HCC tissues compared with normal tissues in analyses of GSE10694 and GSE36915 through DIANA‐mirPath v.3.

KEGG pathway for DEmiRs	*p*
TGF‐beta signaling pathway	4.13E‐05
MAPK signaling pathway	0.000164088
Neurotrophin signaling pathway	0.003509097
Glycosphingolipid biosynthesis—lacto and neolacto series	0.004586994
Ras signaling pathway	0.004586994
Arrhythmogenic right ventricular cardiomyopathy (ARVC)	0.006333444
Hepatitis B	0.009434859

*Note:* Terms with a *p*‐value < 0.05 were considered significant.

### 3.5 miRNA‐mRNA Network Construction in HCC

**FIGURE 9:**
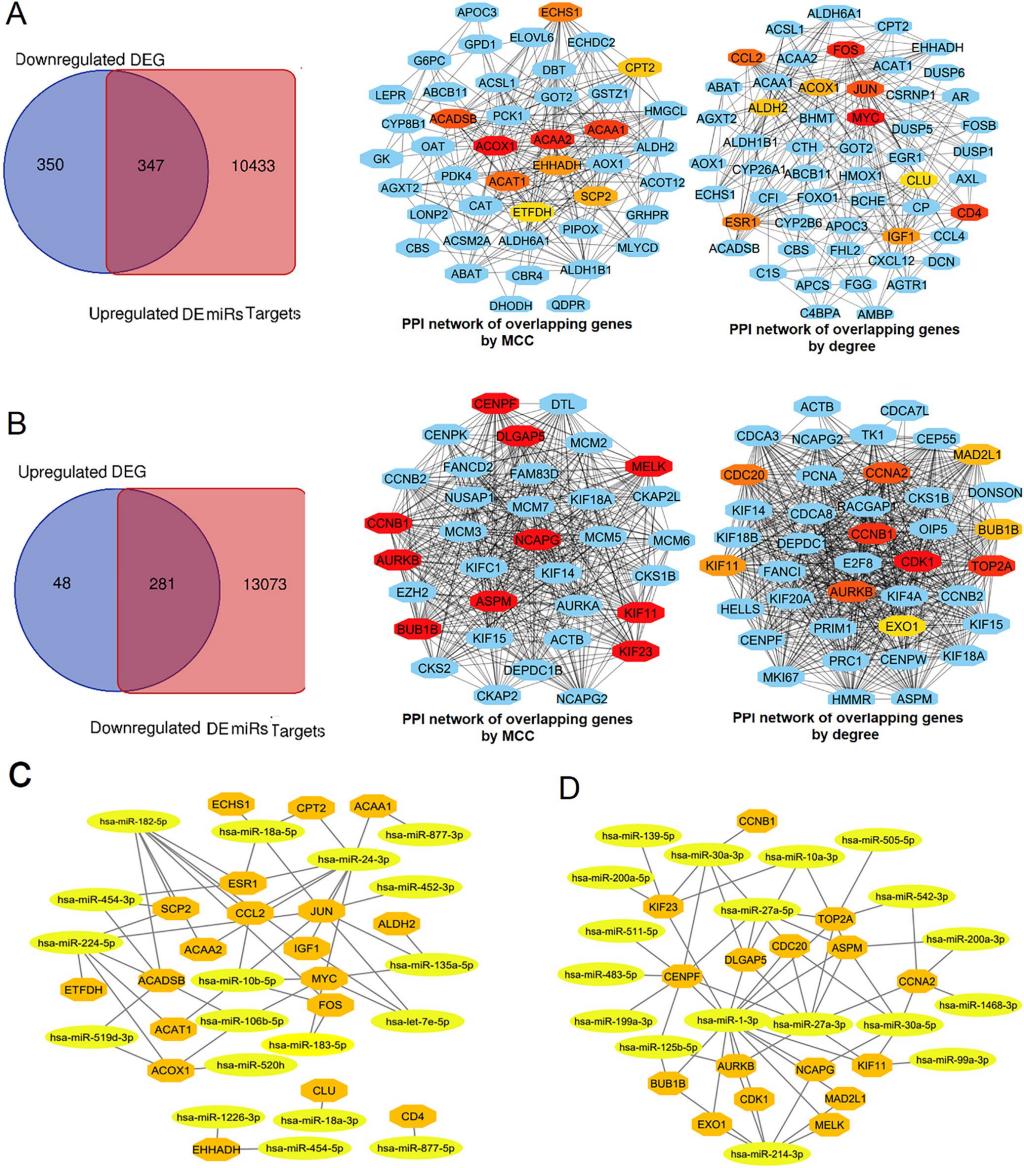
(A) Overlapping genes between predicted target genes of upregulated DEmiRs and decreased DEGs were identified via Venn diagram. PPI networks were then constructed using the overlapping genes. Hub genes in PPI networks were identified by CytoHubba based on the MCC and degree methods. (B) Overlapping genes between predicted targets of downregulated DEmiRs and upregulated DEGs were identified using a Venn diagram, and PPI networks of the obtained overlapping DEGs were generated. Hub genes in PPI networks were identified by CytoHubba based on the MCC and degree methods. (C) The DEmiRs‐DEGs network of decreased hub genes and their related DEmiRs were visualized through Cytoscape software. (D) The DEmiRs‐DEGs network of increased hub genes and their related DEmiRs were visualized through Cytoscape software.

The Multimir package of R software predicted approximately 10 800 target genes for upregulated DEmiRs and 13 300 target genes for downregulated DEmiRs. In cancer, reduced levels of tumor suppressor miRNAs or downregulated DEmiRs may lead to increased levels of oncogenes, while oncomirs or upregulated DEmiRs may result in decreased levels of tumor suppressor genes in cancerous cells. PPI networks were constructed based on the common genes between the target genes of downregulated DEmiRs and increased DEGs, as well as the common genes between the target genes of downregulated DEmiRs and increased DEGs. Using the CytoHubba plugin, increased hub genes and decreased hub genes were obtained (Figure 9A,B). The list of increased hub genes and decreased hub genes is presented in Table 5. Examining the expression of the increased hub genes (using the degree and MCC methods) in the UALKUN dataset confirmed the results of our analysis, as all hub genes showed significantly increased expression. The expression pattern of all downregulated hub genes based on the MCC method was confirmed in the UALKUN database. Additionally, the decreased expression of downregulated hub genes according to the degree method was confirmed in UALKUN, except for the *CCL2* gene (Data are not presented). Considering the importance of hub genes in PPI networks, DEmiRs that can target these genes can be introduced as key miRNAs in HCC.

**TABLE 5:** Increased hub genes and decreased hub genes were identified in PPI network of the up‐regulated overlapping DEG and PPI network of the down‐regulated overlapping DEG, respectively.

Increased hub genes (Target genes of downregulated miRNAs)						Decreased hub genes (Target genes of upregulated miRNAs)					
Ranked by degree			Ranked by MCC			Ranked by degree			Ranked by MCC		
Rank	Name	Score	Rank	Name	Score	Rank	Name	Score	Rank	Name	Score
1	CDK1	131	1	ASPM	6.09E+73	1	MYC	51	1	ACOX1	8 229 241
2	CCNB1	122	1	DLGAP5	6.09E+73	2	FOS	50	2	ACAA2	8 218 351
2	TOP2A	122	1	NCAPG	6.09E+73	3	CD4	46	3	ACAA1	8 188 291
4	AURKB	118	1	KIF23	6.09E+73	4	JUN	45	4	ACADSB	8 186 521
4	CCNA2	118	1	KIF11	6.09E+73	5	CCL2	44	5	ACAT1	8 127 384
6	CDC20	116	1	MELK	6.09E+73	6	ESR1	41	6	ECHS1	8 116 848
7	KIF11	115	1	CCNB1	6.09E+73	7	IGF1	40	7	EHHADH	7 827 920
8	MAD2L1	113	1	BUB1B	6.09E+73	8	ACOX1	39	8	SCP2	7 313 811
8	BUB1B	113	1	AURKB	6.09E+73	9	ALDH2	38	9	CPT2	7 277 305
10	EXO1	112	1	CENPF	6.09E+73	10	CLU	34	10	ETFDH	7 257 608

Finally, the DEmiRs targeting hub genes were identified and presented in Table 6. Based on the predicted DEmiR‐DEGs relationship, DEmiR‐hub genes regulatory networks were obtained and visualized with Cytoscape software (Figure 9C,D). Upregulated miR‐224, miR‐24, and miR‐182 targeted the highest number of downregulated hub genes. Additionally, miR‐1‐3p, miR‐30a, miR‐27a, and miR‐214, which are downregulated miRNAs, potentially targeted 14, 10, 8, and 5 increased hub genes in HCC.

**TABLE 6:** Decreased hub genes and their related DEmiRs, increased hub genes and their related DEmiRs.

Decreased hub genes and their related DEmiRs		Increased hub genes and their related DEmiRs	
Up DEmiRs	Decreased hub genes	Down DEmiRs	Increased hub genes
hsa‐miR‐224‐5p	ACOX1 JUN ACOX1 ACADSB ACAT1 SCP2 ETFDH	hsa‐miR‐1‐3p	CDK1 TOP2A AURKB CDC20 KIF11 MAD2L1 BUB1B EXO1 ASPM DLGAP5 NCAPG KIF23 MELK CENPF
hsa‐miR‐24‐3p	ACOX1 CCL2 ESR1 IGF1 JUN MYC ACAA2 ACAA1 CPT2	hsa‐miR‐30a	CCNB1 CDC20 DLGAP5 KIF23 CENPF CCNA2 CDC20 KIF11 ASPM NCAPG
hsa‐miR‐182‐5p	CCL2 ESR1 FOS IGF1 ACAA2 ACADSB SCP2	hsa‐miR‐27a‐3p	TOP2A AURKB CCNA2 CDC20 KIF11 ASPM DLGAP5 CENPF
hsa‐miR‐106b‐5p	ACOX1 MYC ACOX1 ACADSB	hsa‐miR‐214‐3p	CDK1 AURKB MAD2L1 BUB1B EXO1 NCAPG MELK
hsa‐miR‐10b‐5p	CCL2 FOS JUN MYC ACAT1	hsa‐miR‐27a‐5p	TOP2A ASPM DLGAP5 CENPF
hsa‐miR‐454‐3p	ESR1 ACADSB SCP2 EHHADH	hsa‐miR‐125b‐5p	AURKB BUB1B CENPF
hsa‐miR‐18a‐3p	CLU ESR1 JUN ECHS1 CPT2	hsa‐miR‐200a‐3p	CCNA2 ASPM KIF23
hsa‐miR‐135a‐5p	ALDH2 JUN MYC	hsa‐miR‐10a‐3p	ASPM DLGAP5 KIF23
hsa‐let‐7e‐5p	IGF1 MYC JUN MYC	hsa‐miR‐542‐3p	TOP2A CCNA2

## 4 Discussion

Cancer research is progressing using different approaches with the aim of achieving a correct understanding of cancer and finding effective treatment [54, 55, 56, 57]. The use of computers, bioinformatics analysis, and various software in cancer studies with diverse goals is expanding [8, 58, 59]. In recent years, bioinformatics analysis of transcriptome data has been performed with an emphasis on screening DEGs, identifying key genes, assessing gene expression levels, and conducting survival analysis to identify novel therapeutic targets and molecular markers associated with the diagnosis and prognosis of various malignancies.

In summary, in the present study, DEGs and DEMs were obtained for HCC, hub genes were screened from the PPI network and validated. Genes that may be targets for DEmiRs were predicted using the R multimir package. Eventually, a miRNA–target network was created to present the dysregulated hub miRNAs with more interactions with the hub gene list. Our study, compared with previous similar studies, not only utilized a large sample size of mRNA expression data from the GEO database to identify possible hub genes in HCC, but also investigated the expression profile of non‐coding RNA in HCC to obtain top DEmiRs. Additionally, the miRNA‐mRNA regulation network was obtained through the identification of interactions between the DEmiRs and DEGs.

In this study, we obtained DEGs in five datasets and DEmiRs in two datasets related to HCC About 1000 common DEGs were identified, which were common in at least two or more datasets. We performed GO and KEGG analysis on the overlapping DEGs to gain a deeper understanding of them. The KEGG analysis on overlapping DEGs revealed enrichment in a number of cancer‐related pathways, such as the cell cycle and mitosis.

To understand the connections between DEGs, a PPI network was successfully created, and 20 hub genes were screened through the PPI network using the MCC method. These hub genes include *RRM2*, *MELK*, *KIF11*, *KIF23*, *NCAPG*, *DLGAP5*, *BUB1B*, *AURKB*, *CCNB1*, *KIF20A*, *CCNA2, TTK*, *PBK*, *TOP2A*, *CDK1*, *MAD2L1*, *BIRC5*, *ASPM*, *CDCA8*, and *CENPF*.

Analysis on different GSEs leads to the identification of key genes that may accelerate the understanding of the molecular mechanisms of the disease and provides important therapeutic targets to researchers. Transcriptome analysis in HCC revealed upregulated *ASPM*, *AURKA*, *CCNB2*, *CDC20*, *PRC1*, and *TOP2A* and downregulated *AOX1*, *CAT*, *CYP2E1*, *CYP3A4*, and *HP* as key prognostic genes by GSE36376, GSE39791, GSE57957, and GSE87630 analysis [60]. *CDC20*, *TOP2A*, *ASPM*, *NCAPG*, and *AURKA* were identified as the hub genes in HCC by two gene microarray datasets GSE89377 and GSE101685 analysis [61]. Eleven hub genes *AURKA*, *BUB1B*, *TOP2A*, *MAD2L1*, *CCNA2*, *CCNB1*, *BUB1*, *KIF11*, *CDK1*, *CCNB2*, and *TPX2* were obtained as hub genes by analyze expression profiles GSE101685, GSE62232, GSE46408, and GSE45627 between HCC and normal hepatic tissues [62]. Ten hub genes, *AURKA*, *CDC20*, *FTCD*, *UBE2C*, *CCNB2*, *PTTG1*, *CDKN3*, *CKS1B*, *TOP2A*, and *KIF20A*, were identified as the key genes in HCC that to be significantly correlated with the survival of patients with HCC [63]. The results of the mentioned studies by analysis of the microarray GSEs related to HCC with different bioinformatics methods revealed *CDC20*, *TOP2A*, *ASPM*, *BUB1B*, *MAD2L1*, *KIF20A*, *AURKA*, *CCNA2*, *CCNB1*, *KIF11*, *CDK1*, and *CCNB1* as hub genes in HCC, which is consistent with our study and confirms the importance of these genes in HCC.

Evidence suggests that dysregulation of obtained hub genes is observed in HCC. The expression of *TOP2A* is associated with advanced histological grade in liver cancer [64, 65]. An oncogenic kinase called MELK is required for the early recurrence of HCC [66]. BUB1B promoted the malignancy of HCC. In HCC, BUB1B elevated the mTORC1 signaling pathway [67]. *CDCA8* is essential for chromosomal segregation during mitosis. *CDCA8* knockdown inhibits HCC development and stemness by inactivating oncogenic AKT/β‐catenin signaling and reinstating the ATF3 tumor suppressor [68, 69]. According to recent research, *ASPM* may stimulate cell division and play a role in several human malignancies. By activating the Wnt/β‐catenin pathway, *ASPM* functions as a novel oncoprotein in HCC, thereby facilitating EMT in HCC [70]. Increased expression of *BIRC5*, or survivin, can prevent cells from dying and endow them with the capacity to develop into cancer. In HCC, *BIRC5* expression is upregulated and associated with metastasis and recurrence [71, 72]. *PBK* is essential for controlling DNA damage and repair, as well as cytokinesis. Increased *PBK* expression plays a role in the carcinogenesis or metastasis of HCC [73]. One characteristic of cancer cells is their dysregulation of the cell cycle. The precisely synchronized spindle assembly checkpoint (SAC) is essential for the survival and integrity of normal cells' genome. BUBs, MADs, and TTK are among several SAC proteins that are commonly overexpressed in human malignancies [74]. The G2/M stages of the mammalian cell cycle can be controlled by complexes formed between *CDK1* and cyclin B1 (*CCNB1*) and cyclin B2 (*CCNB2*). In human malignancies, unchecked cell proliferation is linked to CDK dysregulation [75]. Reduced survival in individuals with HCC and breast cancer is associated with abnormal expression of *CCNA2* [76]. *CCNB1* were overexpressed in HCC tissues and the serum of HCC patients [77]. In intracellular transport, KIFs are crucial. In addition to moving chromosomes during mitosis, they also transport protein complexes, membrane organelles, and mRNAs to maintain cellular functions. Upregulation of *KIF20A* is related to malignant behaviors of HCC [78]. According to recent studies, *KIF11* is overexpressed in various cancers and is associated with poor prognoses, including hepatic carcinoma [79]. During mitosis, *KIF23* is essential for the process of cytoplasmic separation. According to current research, *KIF23* expression in HCC patients may be a potential prognostic factor [80, 81]. *NCAPG* is a non‐SMC subunit in the condensin I complex. High expression of this gene has been indicated in HCC [82, 83]. Higher *RRM2* expression is positively related to worse overall survival in HCC [67]. Silencing *AURKA* prevents liver cancer cells from becoming invasive, whereas overexpressing *AURKA* induces epithelial‐mesenchymal transition [84].

The expression of hub genes *RRM2*, *MELK*, *KIF11*, *KIF23*, *NCAPG*, *DLGAP5*, *BUB1B*, *AURKB*, *CCNB1*, *KIF20A*, *CCNA2*, *TTK*, *PBK*, *TOP2A*, *CDK1*, *MAD2L1, BIRC5*, *ASPM*, *CDCA8*, and *CENPF* was validated using GEPIA in HCC. The mRNA expression levels of all hub genes in HCC tissues were higher than in normal tissues, except for *KIF11* and *DLGPA5*, which was consistent with the findings of the microarray analysis. To investigate prognostic markers, we used the Kaplan–Meier plotter to examine the impact of hub gene expression levels on patient survival. High levels of gene expression in all hub genes were associated with poor overall survival in HCC patients. These genes might have a significant impact on HCC development.

miRNA deregulation has been implicated in the pathophysiology of HCC. miRNA controls the expression of target genes by initiating mRNA deterioration, cleaving mRNA, and repressing translation. In the present study, we identified about 60 DEmiRs in HCC of the selected GSE analysis, including the top DEmiRs hsa‐miR‐183, hsa‐miR‐452, hsa‐miR‐886‐5p, hsa‐miR‐551b, hsa‐miR‐96, hsa‐miR‐10b, hsa‐miR‐214*, hsa‐miR‐139‐3p, hsa‐miR‐144*, and hsa‐miR‐125b‐2*, which may serve as useful markers for HCC diagnosis. The role of top DEmiRs in HCC is reviewed in Table 2. Studies confirm the importance of these DEmiRs in HCC. For example miR‐452‐5p might play an important role in HCC as an oncogene and a biomarker [35]. miR‐214 has been suggested as potential biomarker for different cancers [85]. The identification of biomarkers for early tumor diagnosis and patient management is a key topic in current miRNA research by the use of circulating miRNAs. Numerous studies have indicated that serum‐based miRNAs could be helpful as cancer diagnostic markers. In the clinical diagnosis of malignancies, serological markers are easily accessible and obtainable without requiring invasive procedures [37]. For example, it has recently been reported that serum levels of miR‐183 are potential biomarkers for an auxiliary diagnosis in colorectal cancer [86].

The expression patterns of all top DEmiRs were validated in UALKAN according to our results. The miRNA‐pathway analyses mainly identified pathways controlling TGF‐beta signaling and MAPK signaling through the identified DEmiRs. Studies show that these two pathways are involved in HCC. Dysregulated signaling in the TGF‐β pathway influences inflammation, fibrogenesis, and immunomodulation within the HCC microenvironment [87]. In the progression of HCC, TGF‐β and its signaling effectors play opposing roles. Early in the process of carcinogenesis, TGF‐β signaling strongly suppresses tumor growth primarily by cellular senescence, autophagy, apoptosis, and cell cycle arrest. In contrast, TGF‐β functionally switches to a pro‐tumorigenic signal when the tumor advances toward malignancy, instigating aggressive tumor characteristics such as cancer cells' immune evasion, alteration of the tumor microenvironment, and epithelial‐mesenchymal transition. For this reason, inhibiting TGF‐β signaling is recognized as a potential therapy strategy for advanced HCC [88, 89]. In about half of human HCC cases, the MAPK/ERK signaling pathway is active [90].

miRNA are categorized as oncomiRs and tumor suppressors in cancer. Tumor formation and progression are caused by the downregulation of tumor suppressor miRNAs. Tumor growth and cancer progression are accelerated by oncomiRs upregulation [91]. According to the function of miRNA, miRNA that had a decreased expression in HCC samples compared with healthy samples in the analyzes were considered as tumor suppressor miRNAs, whose reduced expression may lead to the elimination of their regulatory effect in the regulation of oncogenes and the formation of cancer. Based on this, increased miRNAs in our analysis were considered as oncomirs or tumor promoting miRNAs. It is possible that their increased expression causes the downregulation of tumor suppressor genes, reducing the expression of tumor suppressor genes facilitates the advancement of processes leading to cancer. To identify key miRNAs in the promotion of HCC, common genes between the decreased DEGs and the targets of the upregulated DEmiRs were isolated. Similarly, common genes between the increased DEGs and the targets of the downregulated DEmiRs were identified. PPI networks were constructed to find hub genes, followed by the identification of DEmiRs targeting these hub genes. Finally, a regulatory axis of miRNA‐mRNA was constructed, offering a framework for future HCC molecular targeted therapy. The two main methods used to create miRNA‐based cancer therapy for regulating miRNA expression. In order to restore the activity of endogenous miRNAs and restore the expression of tumor suppressor miRNAs, synthetic miRNA mimics or miR‐expressing vectors and other small compounds are typically used. Specifically targeting oncomiRs, by miRNA sponges, anti‐miR, and antagomiRs miRNA masking, and small molecule inhibitors, were inhibited overexpressed miRNAs. Several miRNA inhibitors and miRNA mimics are under clinical study in cancer, holding a promise for their therapeutic use [92, 93].

The miRNA‐mRNA network revealed that upregulated miR‐224, miR‐24, and miR‐182 can target 7, 8, and 7 downregulated hub genes, respectively. Additionally, downregulated miRNAs such as miRNA‐1‐3p, miR‐30a, miR‐27a, and miR‐214 potentially target 14, 10, 8, and 7 increased key genes in HCC. The role of miR‐224 was confirmed as an oncomiR in HCC by activating AKT signaling [94]. Upregulation of miR‐224 impacts the promotion of cell migration and invasion [95]. MiR‐24 functions as an oncogene in several cancers; proliferation, migration, and invasion of HCC cells were greatly reduced when miR‐24 was inhibited [96]. A recent report suggests that miR‐224 could be used as a biomarker for early‐stage HCC detection [97]. The expression of miR‐24‐3p was considerably higher in HCC patients compared to healthy controls [98, 99]. MiR‐182 is one of the most significantly up‐regulated miRNAs in HCC and serves as a potential biomarker for diagnosis and therapeutic targeting of HCC [100, 101]. Generally, miRNA‐1‐3p expression is downregulated in HCC cells. Promoting the expression of this miRNA may lead to apoptosis induction and proliferation inhibition. The effect of miRNA‐1‐3p on promoting apoptosis and preventing proliferation may be mediated through the downregulation of SOX9 [102]. Through EMT inhibition, the novel tumor‐suppressor miRNA‐1‐3p dramatically reduces the migration of cancer cells [103]. Tumor suppressor miR‐30a‐3p is downregulated in HCC and prevents tumor growth, invasion, and metastasis [104, 105]. In HCC tissues, miR‐27a‐3p was down‐regulated and associated with metastasis [106]. The tumor suppressive miR‐214 is significantly downregulated in HCC [47, 107, 108]. The role of decreasing and increasing hub genes in the promotion of liver cancer has been determined in different studies. The study's findings provide a more understanding of the fundamental causes of HCC and suggest new biomarkers for diagnosis and prognosis, offering potential treatment approaches for HCC patients. The absence of experimental efforts to verify the outcomes of bioinformatics techniques is the most significant shortcoming of our study. Therefore, further experimental research with larger sample sizes and clinical tissue verification is needed to validate our findings.

## 5 Conclusion

Our study led to the identification of dysregulated genes and miRNAs in HCC through analysis of datasets obtained from the GEO with bioinformatics analysis to identify therapeutic targets and biomarkers in HCC. Gene pathway analysis of the identified hub genes included RRM2, MELK, KIF11, KIF23, NCAPG, DLGAP5, BUB1B, AURKB, CCNB1, KIF20A, CCNA2, TTK, PBK, TOP2A, CDK1, MAD2L1, BIRC5, ASPM, CDCA8, and CENPF revealed their performance in HCC‐related pathways. Top increased DEmiRs included hsa‐miR‐183, hsa‐miR‐452, hsa‐miR‐886‐5p, hsa‐miR‐551b, hsa‐miR‐96, and hsa‐miR‐10b and top decrease DEmiRs included hsa‐miR‐214*, hsa‐miR‐139‐3p, hsa‐miR‐144*, and hsa‐miR‐125b‐2*, were introduced as potential miRNA‐based biomarkers for early tumor diagnosis in HCC. miR‐224, miR‐24, miR‐182 with the possible role of oncomiRs in HCC and miRNA‐1‐3p, miR‐30a, miR‐27a, and miR‐214 with the possible role of tumor suppressor in HCC were identified as key miRNAs with targeting more than six hub genes. The using of miRNA‐based therapies included miRNAs mimics or anti‐miRNAs construct for restoration of suppressed miRNA levels or suppress oncomiRs are developed due to promising results. However, our study had some clear limitations, such as the lack of investigation of liver tissue samples from clinics or reliance solely on the Kaplan–Meier plotter database for prognostic analysis of hub genes. These results provide preliminary data for the identification of new biomarkers and treatment targets for HCC, as well as furthering our understanding of HCC pathogenesis. To confirm the predictive effect of these markers in HCC patients and to investigate the underlying mechanisms or linked pathways, further research is necessary.

## Author Contributions

S.A. Mirzaei coordinated the study, designed the scientific parts, and revised the final manuscript. R. Heidari, V. Assadollahi, S.N. Marashi, and F. Elahian participated in data collection, analyses, discussion, and writing the manuscript in a substantial order. All authors reviewed and accepted the manuscript.

## Ethics Statement

The authors have nothing to report.

## Consent

The authors have nothing to report.

## Conflicts of Interest

The authors declare no conflicts of interest.

## Supporting information


Data S1.

## Data Availability

All databases (including NCBI GEO, Veen Diagram, etc.) are freely available on the web. The raw data of this study are obtained from the GEO database (available at https://www.ncbi.nlm.nih.gov/geo/).
