# Supplementary material for: The miRNA‐mRNA Regulatory Network in Human Hepatocellular Carcinoma by Transcriptomic Analysis From GEO
Source: Cancer Rep (Hoboken). 2025 Jan 7;8(1):e70098. doi: 10.1002/cnr2.70098 (PMC11705453; doi:10.1002/cnr2.70098)
Supplement: Supplementary file 1 — Data S1. [file CNR2-8-e70098-s001.docx]

### **The miRNA-mRNA** [**regulatory network**](https://academic.oup.com/gpb/article-abstract/19/6/913/7230442) **in human hepatocellular carcinoma by transcriptomic analysis from GEO**

**Running title:** Key gene and miRNA in HCC

Razieh Heidari ^1,2^, Vahideh Assadollahi ^3^, Seyedeh Negar Marashi ^2^, Fatemeh Elahian ^2,4^, Seyed Abbas Mirzaei ^2,5*^

1. Cancer Research Center, Basic Health Sciences Institute, Shahrekord University of Medical Sciences, Shahrekord, Iran.
2. Department of Medical Biotechnology, School of Advanced Technologies, Shahrekord University of Medical Sciences, Shahrekord, Iran.
3. Department of Tissue Engineering & Applied Cell Sciences, School of Advanced Technologies, Shahrekord University of Medical Sciences, Shahrekord, Iran.
4. Advanced Technology Cores, Baylor College of Medicine, Texas, USA.
5. Cellular and Molecular Research Center, Basic Health Sciences Institute, Shahrekord University of Medical Sciences, Shahrekord, Iran

Author for Correspondence: Seyed Abbas Mirzaei, PharmD, PhD

Cellular and Molecular Research Center, Basic Health Sciences Institute,

Shahrekord University of Medical Sciences, Shahrekord, Iran

Tel: (+98) 38 33331471; Fax: (+98) 38 33330709

E-mail: mirzaei.a@skums.ac.ir, dr_amirzaei@yahoo.com

**Table 1.** **GO enrichment analysis, Molecular function of obtained overlapping DEGs**.

| Molecular function | No. of genes in the dataset | No. of genes in the background dataset | Percentage of genes | Fold enrichment | P-value (Hypergeometric test) | Bonferroni method | BH method | Genes mapped (from input data set) |
| --- | --- | --- | --- | --- | --- | --- | --- | --- |
| Catalytic activity | 92 | 532 | 10.59908 | 3.611343 | 1.45E-27 | 3.26E-25 | 3.26E-25 | CYP26A1; BBOX1; CYP39A1; ALDH2; ASS1; ADH1C; AKR1D1; ADH4; HAO2; KMO; CYP8B1; ALDH1B1; PCK1; CYP2E1; CYP4A11; ALDH8A1; ADH1B; TDO2; HSD17B2; SRD5A2; ALDH6A1; PANK1; PHGDH; SQLE; CYP2C9; CYP2A7; CA5A; CYP4F12; GCDH; CYP4F2; ENO3; ALPL; AKR1B10; HAO1; CYP2A6; RDH16; CYP1A2; MAT1A; CYP2C8; AKR1C3; CYP3A4; PRODH2; DBH; RDH5; CYP2B6; F11; FMO3; AOX1; HPD; HSD11B1; ACAD11; HPGD; PDK4; ALDH1L1; DHRS1; HGD; CA2; SORD; CYP2J2; MSRA; GPD1; SULF2; HMOX1; TK1; ECHS1; QDPR; AKR1C4; GRHPR; PAH; XDH; MTHFD1; DMGDH; ACADM; MUT; CYP3A43; EHHADH; FASN; COX7B2; CYP4F3; CYP2C18; ADK; ADH6; MTHFD2L; CYP2C19; ACADL; DTYMK; GK; BCO2; GLUD2; NEIL3; GDPD1; PHYHD1; |
| Complement activity | 12 | 28 | 1.382488 | 8.953306 | 2.02E-09 | 4.52E-07 | 2.26E-07 | C9; C7; C8B; C6; C8A; CFI; CRP; CFP; CFHR3; C1R; C1S; C4BPA; |
| Transaminase activity | 9 | 23 | 1.036866 | 8.176392 | 5.66E-07 | 0.000127 | 4.23E-05 | AADAT; GPT2; GPT; TAT; ABAT; AGXT; PSAT1; OAT; GOT2; |
| Protease inhibitor activity | 16 | 94 | 1.843318 | 3.556074 | 9.16E-06 | 0.002051 | 0.000513 | SPP2; SPINK1; SERPINF2; SERPING1; SERPINA11; A2M; SERPINA4; SERPINA7; ITIH1; SERPINC1; CSTB; SERPINB9; SERPINI1; ITIH4; SERPINA1; TFPI2; |
| Extracellular matrix structural constituent | 21 | 166 | 2.419355 | 2.642685 | 4.59E-05 | 0.010278 | 0.002056 | DCN; ECM1; MFAP4; THBS1; CYR61; BGN; RSPO3; COL1A1; COL4A1; COL1A2; LAMC1; ECM2; DPT; PCOLCE; LUM; COL5A2; CTHRC1; CHI3L1; NTN4; ASPN; OLFML3; |
| Protein binding | 22 | 182 | 2.534562 | 2.525098 | 6.14E-05 | 0.013752 | 0.002292 | FGA; GADD45G; CCNB2; MCM6; FGB; GMNN; CDC20; MUC13; SERTAD1; CKS1B; HRG; PEG10; FGG; ANGPTL3; VWF; MAD2L1; CHML; MKI67; SPC25; KIF15; NECAB3; HHIP; |
| Transporter activity | 47 | 576 | 5.414747 | 1.704173 | 0.000264 | 0.059035 | 0.008434 | SHBG; APOF; MT1E; CETP; MT1F; MBL2; MT1M; SLC38A2; MT1X; MTTP; APOA5; NPC1L1; HPX; SLC27A5; APOC4; SAA1; SAA4; HBA2; STARD5; HBB; TTR; HBA1; SLC6A12; FETUB; RBP5; LCN2; HP; SLC25A15; SLC39A14; SLC25A25; SSR2; RAMP3; SPIRE1; RBP4; PEA15; APOC3; SAA2; NUP155; SYTL5; PLSCR4; SLC22A3; GJC1; FLVCR1; SLC23A2; CFHR4; SLC25A47; SLC1A1; |
| Glutathione transferase activity | 6 | 21 | 0.691244 | 5.97313 | 0.000346 | 0.077539 | 0.009692 | GSTZ1; GSTA2; GSTA5; GSTA1; GSTM2; GSTA4; |
| Acyltransferase activity | 12 | 80 | 1.382488 | 3.134384 | 0.000403 | 0.090359 | 0.01004 | NAT2; LCAT; ACAA1; GLYAT; GLYATL1; ACAA2; ACAT1; AGPAT9; PAFAH1B3; DBT; NAT1; MOGAT2; |
| Hydrolase activity | 20 | 203 | 2.304147 | 2.058179 | 0.001821 | 0.407837 | 0.040784 | BCHE; FBP1; EPHX2; NDRG2; UPB1; PON1; GLS2; ARG1; ACOT12; PON3; LYZ; G6PC; DPYS; GCH1; VNN1; FAH; CES1; NEU1; ASPA; MAN1C1; |

**Table 2.** **GO enrichment analysis, Biological process of obtained overlapping DEGs**.

| Biological process | No. of genes in the dataset | No. of genes in the background dataset | Percentage of genes | Fold enrichment | P-value (Hypergeometric test) | Bonferroni method | BH method | Genes mapped (from input data set) |
| --- | --- | --- | --- | --- | --- | --- | --- | --- |
| Energy pathways | 201 | 1633 | 23.15668 | 2.570149 | 7.34E-38 | 1.31E-35 | 1.31E-35 | CYP26A1; BBOX1; CYP39A1; ACSM3; ALDH2; BCHE; ASS1; AKR7A3; ADH1C; NNMT; AKR1D1; ACSL1; FBP1; ADH4; HAO2; GSTZ1; KMO; CYP8B1; ALDH1B1; PCK1; CYP2E1; CYP4A11; AADAT; ACADS; EPHX2; ALDH8A1; INMT; ADH1B; GBA3; TDO2; GPT2; FTCD; HSD17B2; BHMT; SRD5A2; ALDH6A1; NDRG2; PANK1; PHGDH; SQLE; CYP2C9; CYP2A7; CA5A; CYP4F12; GPT; GCDH; SDS; CYP4F2; UPB1; NAT2; AGL; ENO3; DCXR; LIPC; ACACB; LCAT; ACSL4; ALPL; AKR1B10; PON1; HAO1; CYP2A6; ST3GAL6; RDH16; AGXT2; CYP1A2; TAT; ABAT; ACAA1; GLYAT; MAT1A; CYP2C8; PEMT; UGP2; AKR1C3; CYP3A4; SLC27A5; PRODH2; GLS2; MT1A; RDH5; GLYATL1; IYD; CYP2B6; AOX1; ARG1; HPD; ALDOB; UGT2B10; ACAD11; HSD17B13; PNPLA7; SULT1A1; CHST4; SEPP1; HMGCL; ACOT12; UGT2B15; HPGD; GNMT; CSAD; TXNRD1; IDO2; PON3; CBS; ALDH1L1; GNE; CAT; LYZ; CPS1; OTC; G6PC; AGXT; DHRS1; SULT2A1; HGD; NQO1; PSAT1; CA2; SORD; ADH1A; CLYBL; CYP2J2; SHMT1; GCH1; ACAA2; OAT; ACAT1; VNN1; MSRA; GPD1; SULF2; HMOX1; CDA; AGPAT9; TK1; PC; ECHS1; FADS1; UGT2B7; GSTA2; QDPR; AKR1C4; GSTA5; GSTA1; GRHPR; FAH; UGT2B11; PAH; XDH; TKT; HMGCS2; NEU1; MTHFD1; PAFAH1B3; ACLY; DMGDH; ACADM; MUT; CYP3A43; HSD17B6; EHHADH; FASN; COX7B2; CYP4F3; ACSL5; CPT2; CDO1; ETFDH; CYP2C18; ACMSD; ADH6; SAE1; GOT2; SUCLG2; DBT; GSTM2; MTHFD2L; CYP2C19; ACADL; TYMS; ASL; UROC1; HS3ST3B1; GYS2; CHML; DTYMK; NAT1; GGT5; MLYCD; GK; BCO2; GLUD2; GSTA4; ASPA; MAN1C1; MOGAT2; ATAD2; MMAA; PDE11A; PHYHD1; |
| Metabolism | 200 | 1683 | 23.04147 | 2.481387 | 1.94E-35 | 3.45E-33 | 1.72E-33 | CYP26A1; BBOX1; CYP39A1; ACSM3; ALDH2; BCHE; ASS1; AKR7A3; ADH1C; NNMT; AKR1D1; ACSL1; FBP1; ADH4; HAO2; GSTZ1; KMO; CYP8B1; ALDH1B1; PCK1; CYP2E1; CYP4A11; AADAT; ACADS; EPHX2; ALDH8A1; INMT; ADH1B; GBA3; TDO2; GPT2; FTCD; HSD17B2; BHMT; SRD5A2; ALDH6A1; NDRG2; PHGDH; SQLE; CYP2C9; CYP2A7; CA5A; CYP4F12; GPT; GCDH; SDS; CYP4F2; UPB1; NAT2; ENO3; DCXR; LIPC; ACACB; LCAT; ACSL4; ALPL; AKR1B10; PON1; HAO1; CYP2A6; ST3GAL6; RDH16; AGXT2; CYP1A2; TAT; ABAT; ACAA1; GLYAT; MAT1A; CYP2C8; PEMT; UGP2; AKR1C3; CYP3A4; SLC27A5; PRODH2; GLS2; MT1A; RDH5; GLYATL1; IYD; CYP2B6; AOX1; ARG1; HPD; HSD11B1; ALDOB; UGT2B10; ACAD11; HSD17B13; PNPLA7; SULT1A1; CHST4; SEPP1; HMGCL; ACOT12; UGT2B15; HPGD; GNMT; CSAD; TXNRD1; IDO2; PON3; CBS; ALDH1L1; GNE; CAT; LYZ; CPS1; OTC; G6PC; AGXT; DHRS1; SULT2A1; HGD; NQO1; PSAT1; CA2; SORD; ADH1A; CLYBL; CYP2J2; SHMT1; GCH1; ACAA2; OAT; ACAT1; VNN1; MSRA; GPD1; SULF2; HMOX1; CDA; TK1; PC; ECHS1; FADS1; UGT2B7; GSTA2; QDPR; AKR1C4; GSTA5; GSTA1; GRHPR; FAH; UGT2B11; CES1; PAH; XDH; TKT; HMGCS2; NEU1; MTHFD1; PAFAH1B3; ACLY; DMGDH; ACADM; MUT; CYP3A43; HSD17B6; EHHADH; FASN; COX7B2; CYP4F3; ACSL5; CPT2; CDO1; ETFDH; CYP2C18; ACMSD; ADH6; SAE1; GOT2; SUCLG2; DBT; GSTM2; MTHFD2L; CYP2C19; ACADL; TYMS; ASL; UROC1; HS3ST3B1; GYS2; CHML; DTYMK; NAT1; GGT5; MLYCD; GK; BCO2; GLUD2; GSTA4; ASPA; MAN1C1; MOGAT2; ATAD2; MMAA; PDE11A; PHYHD1; |
| Immune response | 49 | 576 | 5.645161 | 1.776578 | 7.19E-05 | 0.012796 | 0.004265 | CD14; HAMP; C9; MBL2; PGLYRP2; C7; C8B; LY6E; FCN3; CLEC4G; C6; C1RL; C8A; FCN2; AZGP1; LECT2; IGLL1; CD34; LEAP2; MARCO; CXCL2; HPR; CFI; CCL14; CRP; CD4; CFP; THY1; CCL20; CFHR3; HP; ORM2; C1R; NFKBIZ; IL4R; CFD; LILRB5; AMBP; C1S; IL1RAP; C4BPA; CCL4; SELE; CFHR4; CCL2; CD5L; MICB; IL6ST; MAGEA1; |
| Xenobiotic metabolism | 3 | 6 | 0.345622 | 10.45735 | 0.001963 | 0.34949 | 0.087372 | NQO1; SULT1A4; UGT1A6; |
| Lipid transport | 3 | 7 | 0.345622 | 8.965573 | 0.003314 | 0.589872 | 0.117974 | SAA1; STARD5; PLIN2; |
| Wound healing | 2 | 3 | 0.230415 | 13.94309 | 0.006654 | 1 | 0.197417 | IGF1; ANGPTL6; |
| Cell cycle | 4 | 22 | 0.460829 | 3.804117 | 0.019179 | 1 | 0.431897 | PRC1; NUSAP1; CDC20; ZWINT; |
| Regulation of metabolism | 2 | 5 | 0.230415 | 8.376988 | 0.020799 | 1 | 0.431897 | GDPD1; NECAB3; |
| DNA replication | 3 | 13 | 0.345622 | 4.830797 | 0.021838 | 1 | 0.431897 | MCM7; MCM8; RFC3; |
| Chemosensory behavior | 1 | 1 | 0.115207 | 20.87996 | 0.047892 | 1 | 0.743362 | CCL15; |

**Table 3.** **GO enrichment analysis, Biological pathway of obtained overlapping DEGs**.

| Biological pathway | No. of genes in the dataset | No. of genes in the background dataset | Percentage of genes | Fold enrichment | P-value (Hypergeometric test) | Bonferroni method | BH method | Q-value (Storey-Tibshirani method) | Genes mapped (from input data set) |
| --- | --- | --- | --- | --- | --- | --- | --- | --- | --- |
| Cell Cycle, Mitotic | 65 | 317 | 12.62136 | 2.504626 | 1.06E-12 | 1.77E-09 | 1.77E-09 | 6.74875E-09 | PTTG1; UBE2C; TOP2A; PSMD4; AURKA; CCNB2; MCM2; NEK2; MCM6; MCM3; GMNN; CDC20; AURKB; CKS1B; PCNA; RFC4; KIF20A; CDC25B; MCM7; CENPM; HSP90AA1; CCNE1; TUBA1A; MCM4; RPS27; CENPQ; CCNB1; GINS1; BIRC5; KNTC1; CDK1; MAD2L1; PRIM2; CENPJ; CENPL; TYMS; MCM5; ZWINT; CCNA2; KIF23; CENPE; CCNE2; CDKN2C; CENPK; MCM8; RFC3; CDC25C; ZWILCH; CENPO; ITGB3BP; PRKAR2B; PRIM1; CASC5; RRM2; FEN1; TUBG1; SPC25; KIF18A; BUB1B; CEP41; SGOL2; CEP152; CDKN2A; CENPF; CDCA8; |
| Biological oxidations | 32 | 98 | 6.213592 | 3.98888 | 2.58E-12 | 4.3E-09 | 2.15E-09 | 8.20854E-09 | CYP26A1; CYP39A1; ALDH2; NNMT; CYP8B1; CYP2E1; CYP4A11; CYP2C9; CYP4F2; NAT2; CYP2A6; CYP1A2; GLYAT; CYP2C8; UGP2; CYP3A4; CYP2B6; FMO3; SULT1A1; SULT2A1; CYP2J2; SULT1A4; UGT1A6; GSTA2; GSTA5; GSTA1; CYP3A43; CYP4F3; CYP2C18; CYP2C19; NAT1; GSTA4; |
| Metabolism of amino acids and derivatives | 42 | 188 | 8.15534 | 2.729024 | 9.32E-10 | 1.56E-06 | 5.11E-07 | 1.94955E-06 | BBOX1; ASS1; GSTZ1; KMO; AADAT; UBE2C; TDO2; GPT2; FTCD; ALDH6A1; GPT; GCDH; PSMD4; HAO1; AGXT2; TAT; DBH; IYD; ARG1; HPD; IDO2; AMDHD1; CPS1; OTC; AGXT; HGD; NQO1; SHMT1; SLC25A15; OAT; ACAT1; QDPR; GRHPR; FAH; PAH; ASNS; ACMSD; GOT2; DBT; ADI1; ASL; UROC1; |
| Metabolism of lipids and lipoproteins | 51 | 257 | 9.902913 | 2.424047 | 1.22E-09 | 2.04E-06 | 5.11E-07 | 1.94955E-06 | CYP39A1; AKR1D1; ACSL1; CETP; CYP8B1; SLCO1B3; CYP4A11; ACADS; SRD5A2; SQLE; SLC10A1; LIPC; ACACB; LCAT; ACSL4; MTTP; APOA5; NPC1L1; ACAA1; SLC27A5; STARD5; PLIN2; ZFP36; HSD11B1; A2M; HMGCL; TXNRD1; SLC2A2; SULT2A1; SLCO1B1; ACAT1; PPAP2B; ELOVL6; GPD1; ECHS1; FADS1; AKR1C4; HMGCS2; ACLY; ACADM; MUT; SLC27A2; FASN; ACSL5; CPT2; APOC3; ACADL; LDLR; GGT5; GK; ELOVL7; |
| Fatty acid, triacylglycerol, and ketone body metabolism | 25 | 83 | 4.854369 | 3.679755 | 4.53E-09 | 7.56E-06 | 1.51E-06 | 5.77343E-06 | ACSL1; CYP4A11; ACADS; ACACB; ACSL4; PLIN2; HMGCL; TXNRD1; SLC2A2; SULT2A1; ACAT1; ELOVL6; GPD1; ECHS1; FADS1; HMGCS2; ACLY; ACADM; MUT; FASN; ACSL5; CPT2; ACADL; GK; ELOVL7; |
| DNA Replication | 50 | 261 | 9.708738 | 2.340105 | 6.31E-09 | 1.05E-05 | 1.76E-06 | 6.7005E-06 | UBE2C; PSMD4; MCM2; NEK2; MCM6; MCM3; GMNN; CDC20; AURKB; PCNA; RFC4; KIF20A; MCM7; CENPM; HSP90AA1; TUBA1A; MCM4; RPS27; CENPQ; GINS1; BIRC5; KNTC1; CDK1; MAD2L1; PRIM2; CENPJ; CENPL; MCM5; ZWINT; KIF23; CENPE; CENPK; MCM8; RFC3; ZWILCH; CENPO; ITGB3BP; PRKAR2B; PRIM1; CASC5; FEN1; TUBG1; SPC25; KIF18A; BUB1B; CEP41; SGOL2; CEP152; CENPF; CDCA8; |
| Complement cascade | 12 | 22 | 2.330097 | 6.664363 | 2.42E-08 | 4.04E-05 | 5.77E-06 | 2.20127E-05 | C9; MBL2; C7; C8B; C6; C8A; MASP2; MASP1; CRP; C1R; CFD; C1S; |
| Metabolism | 110 | 823 | 21.35922 | 1.632536 | 3.5E-08 | 5.84E-05 | 6.82E-06 | 2.60437E-05 | CYP26A1; BBOX1; CYP39A1; ALDH2; ASS1; NNMT; GSTZ1; KMO; CYP8B1; CYP2E1; CYP4A11; AADAT; UBE2C; TDO2; GPT2; FTCD; ALDH6A1; PANK1; CYP2C9; GCGR; GPT; GCDH; CYP4F2; UPB1; NAT2; PSMD4; HAO1; CYP2A6; SLC19A3; AGXT2; CYP1A2; TAT; GLYAT; CYP2C8; UGP2; CYP3A4; DBH; ZFP36; IYD; CYP2B6; FMO3; ARG1; HPD; TPR; SULT1A1; TXNRD1; IDO2; PDK4; AMDHD1; CAT; CPS1; OTC; AGXT; SULT2A1; DPYS; HGD; NQO1; CYP2J2; SHMT1; HSPB1; GCH1; SLC25A15; OAT; ACAT1; SULT1A4; RPLP0; HSP90AA1; HMOX1; CDA; UGT1A6; TK1; GSTA2; QDPR; YWHAZ; GSTA5; GSTA1; GRHPR; FAH; PAH; XDH; TKT; MTHFD1; ACLY; CYP3A43; FASN; CYP4F3; ETFDH; CYP2C18; ADK; ASNS; ACMSD; SNRPB; GOT2; SUCLG2; DBT; RPS27; ADI1; NUP155; CYP2C19; TYMS; ASL; NAMPT; UROC1; DTYMK; NAT1; SLC23A2; GSTA4; PRKAR2B; RRM2; DHODH; |
| DNA strand elongation | 14 | 31 | 2.718447 | 5.517877 | 3.68E-08 | 6.14E-05 | 6.82E-06 | 2.60437E-05 | MCM2; MCM6; MCM3; PCNA; RFC4; MCM7; MCM4; GINS1; PRIM2; MCM5; MCM8; RFC3; PRIM1; FEN1; |
| FOXM1 transcription factor network | 16 | 42 | 3.106796 | 4.654514 | 7.26E-08 | 0.000121 | 1.21E-05 | 4.62476E-05 | FOS; ESR1; CCNB2; NEK2; AURKB; CKS1B; CDC25B; CCNE1; MAP2K1; CCNB1; BIRC5; FOXM1; CDK1; CCNA2; CDKN2A; CENPF; |

**Table 4.** **GO enrichment analysis, Cellular component of obtained overlapping DEGs**.

| GO enrichment: Cellular component | | | | | | | | |
| --- | --- | --- | --- | --- | --- | --- | --- | --- |
| Cellular component | **No. of genes in the dataset** | **No. of genes in the background dataset** | **Percentage of genes** | **Fold enrichment** | **P-value (Hypergeometric test)** | **Bonferroni method** | **BH method** | **Genes mapped (from input data set)** |
| Extracellular | 205 | 1825 | 26.2484 | 2.093177 | 8.73E-27 | 6.84E-24 | 6.84E-24 | IGF1; GHR; SHBG; BCHE; KLKB1; APOF; CD14; CETP; PLG; GPC3; IGFBP3; HAMP; DCN; MT2A; C9; PROZ; MBL2; F9; IGFALS; EGR1; ECM1; PGLYRP2; MT1X; C7; C8B; FGA; CXCL12; MDK; INMT; FCN3; C6; ALDH6A1; PHGDH; SQLE; GCGR; GPT; ANXA2; ANGPTL4; AGL; ENO3; SPP2; LIPC; LCAT; HGFAC; ALPL; SPINK1; C8A; PON1; FCN2; APOA5; AFM; SERPINF2; TAT; AZGP1; LECT2; HPX; SERPING1; DBH; SAA1; SAA4; CD34; MFAP4; LEAP2; PLIN2; CRHBP; CCNB2; MASP2; THBS1; OIT3; SRPX; CYR61; PLAC8; BGN; CXCL2; MASP1; F11; HBB; RNASE4; FGB; HPR; ARG1; ANG; TTR; CFI; APCS; A2M; ASGR2; SEPP1; HMGCL; HBA1; HABP2; REG3A; ANGPTL6; CCL14; TXNRD1; CRP; PDGFRA; RSPO3; BASP1; S100A8; NEB; CFP; RELN; HRG; PON3; COL1A1; FETUB; SPP1; SERPINA4; HSP90AB1; COL4A1; CAT; LYZ; COL1A2; LEPR; CCL20; MST1; LAMC1; SERPINA7; IGFBP1; CA2; ITIH1; LCN2; HP; FGG; ORM2; SLCO1B1; C1R; ANGPTL3; CD163; ECM2; AR; DPT; CCL15; F7; ACTB; PRSS8; PCOLCE; OXT; SULF2; CDA; STC1; CCL19; SVEP1; LUM; AXL; STAB1; IL4R; CFD; ESM1; ITLN1; IGFBP4; QDPR; AHSG; DLK1; GSTA1; AMBP; RBP4; FAH; F12; CES1; XDH; TKT; PAFAH1B3; COL5A2; C1S; MUT; VWF; DKK1; SERPINC1; C4BPA; CTHRC1; RPS27; APOC3; SAA2; CHI3L1; JUND; KIF4A; NTN4; CCL4; KIF23; ASPN; ASL; NAMPT; DLGAP5; OLFML3; LRG1; ITIH4; HGF; EZH2; SERPINA1; IL1RL1; SELE; CFHR4; CCL2; ADAMTS1; CD5L; CKAP2; RRM2; A1BG; IL6ST; MSH2; ADAMTS13; STC2; TFPI2; |
| Extracellular region | 79 | 442 | 10.11524 | 3.330782 | 9E-22 | 7.05E-19 | 3.53E-19 | IGF1; SHBG; BCHE; KLKB1; APOF; CD14; CETP; PLG; IGFBP3; HAMP; MBL2; F9; IGFALS; C7; C8B; FGA; FCN3; C6; ANGPTL4; LCAT; HGFAC; C8A; PON1; APOA5; SERPINF2; AZGP1; HPX; SERPING1; DBH; SAA1; SAA4; PLIN2; MASP2; THBS1; MASP1; F11; FGB; TTR; APCS; A2M; SEPP1; HABP2; CRP; CFP; HRG; PON3; COL1A1; FETUB; SPP1; COL4A1; COL1A2; LAMC1; SERPINA7; ITIH1; HP; FGG; C1R; CD163; F7; CDA; CCL19; LUM; CFD; ESM1; AHSG; AMBP; C1S; VWF; SERPINC1; C4BPA; APOC3; CCL4; LRG1; ITIH4; HGF; SERPINA1; CCL2; CD5L; A1BG; |
| Extracellular space | 66 | 404 | 8.450704 | 3.044485 | 2.85E-16 | 2.24E-13 | 7.45E-14 | IGF1; GHR; KLKB1; PRC1; CETP; PLG; IGFBP3; DCN; MBL2; IGFALS; FGA; ANGPTL4; LIPC; LCAT; C8A; PON1; APOA5; LECT2; HPX; THBS1; CXCL2; MASP1; F11; FGB; ANG; APCS; HABP2; REG3A; CCL14; CFP; COL1A1; SERPINA4; LYZ; COL1A2; CCL20; LAMC1; SERPINA7; IGFBP1; SORD; CFHR3; FGG; ORM2; ANGPTL3; DPT; CCL15; HSP90AA1; PRSS8; PCOLCE; SULF2; HMOX1; LUM; AHSG; DLK1; RBP4; F12; SERPINC1; APOC3; SERPINB9; CHI3L1; CCL4; SERPINA1; SELE; CCL2; HMGB2; CD5L; IL6ST; |
| Exosomes | 191 | 2043 | 24.45583 | 1.742135 | 1.01E-15 | 7.88E-13 | 1.97E-13 | BBOX1; ALDH2; ASS1; AKR7A3; CD14; FBP1; CETP; PLG; C1QTNF1; C9; PROZ; MBL2; IGFALS; ECM1; PCK1; EPHX2; FGA; ALDH8A1; FCN3; CLRN3; FTCD; BHMT; SLC39A5; NDRG2; PHGDH; GPT; ANXA2; ANGPTL4; UPB1; ENO3; DCXR; ACSL4; ALPL; SPINK1; AKR1B10; PON1; FCN2; ST3GAL6; CTH; AZGP1; S100A10; UGP2; HPX; SERPING1; SAA1; HBA2; PLIN2; RACGAP1; MASP2; TACSTD2; THBS1; TMEM27; COLEC10; RHOB; BGN; MASP1; F11; HBB; AOX1; FGB; HPR; HPD; TTR; CFI; APCS; ALDOB; A2M; TUBA1C; CCT3; STOM; SEPP1; HBA1; SORL1; MUC13; HPGD; TXNRD1; CYFIP2; MXRA5; PLVAP; BASP1; PCNA; S100A8; NEB; RELN; HRG; PON3; THY1; SERPINA4; ALDH1L1; HSP90AB1; LYZ; HRSP12; RBP5; S100P; TUBA1B; DPYS; LAMC1; CD24; HGD; SERPINA7; NQO1; PSAT1; CA2; SORD; CFHR3; LCN2; CYP2J2; HP; FGG; SHMT1; HSPB1; MME; ACAA2; SLC46A3; C1R; ACAT1; MSRA; IFITM1; F7; RPLP0; HSP90AA1; ACTB; PRSS8; GPD1; LGALS4; FAT1; SLC22A11; CUX2; CFD; ITLN1; PROM1; GSTA2; QDPR; YWHAZ; TUBA1A; AHSG; GSTA1; AMBP; GRHPR; RBP4; FAH; XDH; TKT; NEU1; PBLD; ATP6V1C1; MTHFD1; ACLY; PODXL; SLC27A2; FASN; VWF; ADK; ASNS; ADH6; GOT2; C4BPA; RHEB; CSTB; GSTM2; RAB17; SAA2; IGSF3; SERPINB9; CDK1; NEDD4L; ITGA6; PRKDC; GPM6A; STMN1; ABCC9; ASL; NAMPT; SLC4A4; ITIH4; COBLL1; GK; KIF18B; SERPINA1; SMC2; PLCB1; ATAD2; CD5L; SLC1A1; FIGNL1; HTATIP2; EHD3; A1BG; KIF15; NDRG3; CDHR2; |
| Microsome | 28 | 156 | 3.585147 | 3.345468 | 1.51E-08 | 1.19E-05 | 2.37E-06 | CYP26A1; CYP39A1; ASS1; CYP2E1; CYP4A11; SQLE; CYP2C9; CYP2A7; ACSL4; CYP2A6; RDH16; CYP1A2; CYP2C8; CYP3A4; CYP2B6; FMO3; NQO1; CYP2J2; SSR2; ELOVL6; HMOX1; UGT1A6; FADS1; SLC27A2; CYP2C19; PRKDC; ABCC9; MOGAT2; |
| Microtubule | 25 | 129 | 3.201024 | 3.612323 | 1.82E-08 | 1.43E-05 | 2.38E-06 | PRC1; ASPM; RACGAP1; TUBA1C; CCT3; KIFC1; FAM83D; KIF20A; HSP90AB1; HSPB1; HSP90AA1; TUBA1A; TBCE; TPX2; BIRC5; CDK1; KIF14; KIF4A; BARD1; KIF23; CENPE; DLGAP5; KIF11; CKAP2; KIF18A; |
| Platelet alpha granule lumen | 12 | 35 | 1.536492 | 6.392153 | 1.39E-07 | 0.000109 | 1.56E-05 | IGF1; FGA; SERPINF2; SERPING1; THBS1; FGB; A2M; HRG; FGG; CFD; HGF; SERPINA1; |
| Endoplasmic reticulum membrane | 25 | 150 | 3.201024 | 3.106631 | 3.91E-07 | 0.000307 | 3.83E-05 | CYP26A1; CYP39A1; CYP8B1; CYP2E1; CYP4A11; HSD17B2; SQLE; CYP2C9; CYP4F12; CYP4F2; CYP2A6; CYP1A2; CYP2C8; CYP3A4; SLC27A5; CYP2B6; FMO3; HSD11B1; CD4; G6PC; AGPAT9; CYP4F3; CYP2C18; CYP2C19; NECAB3; |
| Cytosol | 102 | 1178 | 13.06018 | 1.613578 | 7.23E-07 | 0.000567 | 6.3E-05 | ASS1; AKR7A3; PTTG1; AKR1D1; ACSL1; GSTZ1; KMO; PCK1; EPHX2; UBE2C; GBA3; TDO2; NDRG2; PANK1; PHGDH; UPB1; NAT2; ACACB; STARD5; ZFP36; FOXO1; CCNB2; TACSTD2; NEK2; CIDEB; HPD; A2M; CCT3; CDC20; HPGD; TXNRD1; CBS; GNE; HSP90AB1; AMDHD1; TUBA1B; SULT2A1; DPYS; PSAT1; SHMT1; CDC25B; GCH1; SULT1A4; DNMT3L; CAMK2B; SYT7; RPLP0; HSP90AA1; ACTB; GPD1; SPRY2; LGALS4; HMOX1; GCKR; CDA; SRXN1; CCNE1; YWHAZ; AKR1C4; TUBA1A; FAH; TKT; MTHFD1; ACLY; SIK1; FASN; CDO1; ASNS; ACMSD; SNRPB; MAP2K1; RAB3IP; RHEB; RPS27; CCNB1; BIRC5; SERPINB9; CDK1; FXN; MAD2L1; CENPJ; CENPL; FYN; PRKDC; PSRC1; NAMPT; DTYMK; CCNE2; SPTBN2; S100A12; NAT1; GK; CDC25C; ENAH; ITGB3BP; PRKAR2B; PLCB1; EIF5; TUBG1; BUB1B; CEP41; CEP152; |
| Mitochondrion | 103 | 1259 | 13.18822 | 1.524567 | 8.21E-06 | 0.00644 | 0.000644 | ACSM3; ALDH2; ACSL1; KMO; ALDH1B1; PCK1; AADAT; ACADS; ACSM5; INMT; ADH1B; GPT2; HSD17B2; ALDH6A1; CA5A; GPT; GCDH; DCXR; ACACB; ACSL4; TAT; ABAT; ACAA1; GLYAT; OGDHL; PRODH2; GLS2; MT1A; AURKA; CIDEB; MCM3; HMGCL; RAD51; SLC25A18; AURKB; CKS1B; UHRF1; PDK4; HSP90AB1; CAT; CPS1; OTC; AGXT; TUBA1B; NQO1; SHMT1; ACAA2; SLC25A15; OAT; ACAT1; SLC25A25; MSRA; HMMR; PMPCA; NTHL1; RPLP0; HSP90AA1; ACTB; GPD1; GPAA1; PC; ECHS1; DNMT1; HMGCS2; MTHFD1; ACLY; DMGDH; ACADM; MUT; EHHADH; FASN; COX7B2; ACSL5; CPT2; ETFDH; GOT2; SUCLG2; DBT; NUP155; CDK1; ACADL; FXN; KIF4A; PRKDC; TYMS; MCM5; NR4A1; CASP4; SATB1; MLYCD; FANCD2; GK; GLUD2; GSTA4; SMC4; ASPA; HMGB2; SLC25A47; SMC2; MMAA; FEN1; FANCI; DHODH; |

**Table 5. DEmiRs in HCC tissues compared with normal tissues were identified in analyses of GSE36915 based on a P-Value <0.05 and |log2FC|>1. (FC: fold-change, DEmiR: Differentially expressed miRNA, HCC: hepatocellular carcinoma).**

| **SPOT_ID** | **logFC** | **P.Value** | **adj.P.Val** |
| --- | --- | --- | --- |
| hsa-miR-183 | 2.706142 | 8.65E-11 | 1.98E-08 |
| hsa-miR-452 | 2.026533 | 3.55E-09 | 2.26E-07 |
| hsa-miR-886-5p | 1.749826 | 1.01E-07 | 3.13E-06 |
| hsa-miR-551b | 1.667185 | 1.40E-03 | 5.72E-03 |
| hsa-miR-96 | 1.661249 | 2.76E-06 | 3.72E-05 |
| hsa-miR-10b | 1.576938 | 1.01E-04 | 0.000691 |
| hsa-miR-190b | 1.547697 | 3.57E-06 | 4.50E-05 |
| hsa-miR-877 | 1.514522 | 5.20E-10 | 5.41E-08 |
| hsa-miR-452*:9.1 | 1.496605 | 3.30E-06 | 4.20E-05 |
| hsa-miR-1180 | 1.479568 | 1.85E-08 | 8.82E-07 |
| hsa-miR-135a | 1.476325 | 7.57E-05 | 5.48E-04 |
| hsa-miR-182 | 1.465659 | 9.96E-07 | 1.73E-05 |
| hsa-miR-224 | 1.416474 | 2.12E-05 | 0.000189 |
| hsa-miR-515-5p | 1.410513 | 0.012864 | 0.035837 |
| hsa-miR-520h, hsa-miR-520g | 1.392929 | 0.013809 | 0.0381 |
| hsa-miR-501-5p | 1.333418 | 3.95E-10 | 4.90E-08 |
| hsa-miR-106b* | 1.326271 | 1.13E-10 | 2.16E-08 |
| hsa-miR-454* | 1.288562 | 2.77E-09 | 1.86E-07 |
| hsa-miR-1226 | 1.218099 | 1.11E-07 | 3.35E-06 |
| hsa-miR-519d | 1.152403 | 1.63E-02 | 0.043804 |
|  | 1.151068 | 3.05E-06 | 4.02E-05 |
| hsa-miR-516b | 1.150062 | 0.02544 | 0.061976 |
| hsa-miR-1269 | 1.146963 | 7.81E-05 | 0.000562 |
| hsa-miR-1303 | 1.145746 | 3.69E-08 | 1.53E-06 |
| hsa-miR-217 | 1.125249 | 0.016665 | 0.044376 |
| hsa-miR-15b* | 1.110346 | 5.36E-08 | 1.92E-06 |
| hsa-miR-518e*, hsa-miR-519a*, hsa-miR-519b-5p, hsa-miR-519c-5p, hsa-miR-522*, hsa-miR-523* | 1.091807 | 0.030246 | 0.072149 |
| hsa-miR-518f | 1.090381 | 0.032945 | 0.077458 |
| hsa-miR-216a | 1.064357 | 0.004095 | 0.014039 |
| hsa-miR-431 | 1.055786 | 0.009309 | 0.027451 |
| hsa-miR-34b* | 1.047345 | 8.50E-07 | 1.61E-05 |
| hsa-miR-520g | 0.991803 | 0.015143 | 0.041088 |
| hsa-miR-144:9.1 | -1.00606 | 0.002606 | 0.009596 |
| hsa-miR-99a* | -1.02554 | 4.11E-12 | 2.36E-09 |
|  | -1.04768 | 3.90E-10 | 4.90E-08 |
| hsa-miR-542-3p | -1.05591 | 1.08E-05 | 0.00011 |
| hsa-miR-511 | -1.05627 | 6.66E-06 | 7.56E-05 |
| hsa-miR-505 | -1.05851 | 2.55E-05 | 2.21E-04 |
| hsa-miR-200a | -1.10648 | 0.009253 | 0.027377 |
| hsa-miR-1468 | -1.12038 | 0.001428 | 0.005776 |
| hsa-miR-199a*:9.1 | -1.13343 | 1.38E-06 | 2.20E-05 |
| hsa-miR-429 | -1.14191 | 0.007287 | 0.022489 |
| hsa-miR-136 | -1.1453 | 0.006767 | 0.021229 |
| hsa-miR-30a* | -1.15153 | 5.92E-07 | 1.19E-05 |
| hsa-miR-1 | -1.16146 | 8.55E-06 | 9.15E-05 |
| hsa-miR-10a | -1.26524 | 9.47E-06 | 0.0001 |
| hsa-miR-483-3p | -1.28598 | 0.011633 | 0.033134 |
| hsa-miR-378* | -1.31597 | 2.18E-09 | 1.67E-07 |
| hsa-miR-139-5p | -1.33025 | 4.28E-10 | 4.90E-08 |
| hsa-miR-375 | -1.47175 | 1.45E-03 | 0.005836 |
| hsa-miR-203 | -1.47431 | 0.000277 | 0.001511 |
| hsa-miR-125b-2* | -1.53818 | 9.69E-09 | 5.28E-07 |
| hsa-miR-144* | -1.54111 | 6.19E-06 | 7.16E-05 |
| hsa-miR-139-3p | -1.66415 | 5.58E-13 | 6.39E-10 |
| hsa-miR-214* | -1.86906 | 3.75E-08 | 1.53E-06 |

**Table 6. DEmiRs in HCC tissues compared with normal tissues were identified in analyses of GSE10694 based on a P-Value <0.05 and |log2FC|>1. (FC: fold-change, DEmiR: Differentially expressed miRNA, HCC: hepatocellular carcinoma).**

| SPOT_ID | logFC | P.Value | adj.P.Val |
| --- | --- | --- | --- |
| hsa-miR-18a | 1.667659 | 5.00E-23 | 6.04E-21 |
| hsa-let-7e | 1.527954 | 6.16E-21 | 3.73E-19 |
| hsa-miR-523 | 1.0999 | 1.77E-15 | 5.37E-14 |
| hsa-miR-24 | 1.069566 | 3.56E-14 | 6.15E-13 |
| hsa-miR-27a | -1.02492 | 1.35E-10 | 1.36E-09 |
